# Middle and Late Pleistocene Denisovan subsistence at Baishiya Karst Cave

**DOI:** 10.1038/s41586-024-07612-9

**Published:** 2024-07-03

**Authors:** Huan Xia, Dongju Zhang, Jian Wang, Zandra Fagernäs, Ting Li, Yuanxin Li, Juanting Yao, Dongpeng Lin, Gaudry Troché, Geoff M. Smith, Xiaoshan Chen, Ting Cheng, Xuke Shen, Yuanyuan Han, Jesper V. Olsen, Zhongwei Shen, Zhiqi Pei, Jean-Jacques Hublin, Fahu Chen, Frido Welker

**Affiliations:** 1 Key Laboratory of Western China’s Environmental Systems (Ministry of Education), Key Scientific Research Base of Bioarchaeology in Cold and Arid Regions (National Cultural Heritage Administration), College of Earth and Environmental Sciences, Lanzhou University, Lanzhou, China; 2 Alpine Paleoecology and Human Adaptation Group (ALPHA), State Key Laboratory of Tibetan Plateau Earth System, Environment and Resources (TPESER), Institute of Tibetan Plateau Research (ITPCAS), Chinese Academy of Sciences (CAS), Beijing, China; 3 College of Ecology, Lanzhou University, Lanzhou, China; 4 School of Earth Sciences, Lanzhou University, Lanzhou, China; 5 Globe Institute, University of Copenhagen, Copenhagen, Denmark; 6 School of Anthropology and Conservation, University of Kent, Canterbury, UK; 7 Department of Archaeology, University of Reading, Reading, UK; 8 Novo Nordisk Foundation Center for Protein Research, University of Copenhagen, Copenhagen, Denmark; 9 Gansu Provincial Museum, Lanzhou, China; 10 Chaire de Paléoanthropologie, CIRB, Collège de France, Université PSL, CNRS, Paris, France; 11 Max Planck Institute for Evolutionary Anthropology, Leipzig, Germany; 12 University of Chinese Academy of Sciences, Beijing, China

## Abstract

Genetic and fragmented palaeoanthropological data suggest that Denisovans were once widely distributed across eastern Eurasia^1–3^. Despite limited archaeological evidence, this indicates that Denisovans were capable of adapting to a highly diverse range of environments. Here we integrate zooarchaeological and proteomic analyses of the late Middle to Late Pleistocene faunal assemblage from Baishiya Karst Cave on the Tibetan Plateau, where a Denisovan mandible and Denisovan sedimentary mitochondrial DNA were found^3,4^. Using zooarchaeology by mass spectrometry, we identify a new hominin rib specimen that dates to approximately 48–32 thousand years ago (layer 3). Shotgun proteomic analysis taxonomically assigns this specimen to the Denisovan lineage, extending their presence at Baishiya Karst Cave well into the Late Pleistocene. Throughout the stratigraphic sequence, the faunal assemblage is dominated by Caprinae, together with megaherbivores, carnivores, small mammals and birds. The high proportion of anthropogenic modifications on the bone surfaces suggests that Denisovans were the primary agent of faunal accumulation. The *chaîne opératoire* of carcass processing indicates that animal taxa were exploited for their meat, marrow and hides, while bone was also used as raw material for the production of tools. Our results shed light on the behaviour of Denisovans and their adaptations to the diverse and fluctuating environments of the late Middle and Late Pleistocene of eastern Eurasia.

## Abstract (web summary)

Zooarchaeological and proteomic analyses of bones from Baishiya Karst Cave on the Tibetan Plateau identify a hominin rib specimen, and provide insight into the ways Denisovans interacted with their surrounding environment and made use of animal resources.

## Main

**Extended Data Fig. 1:**
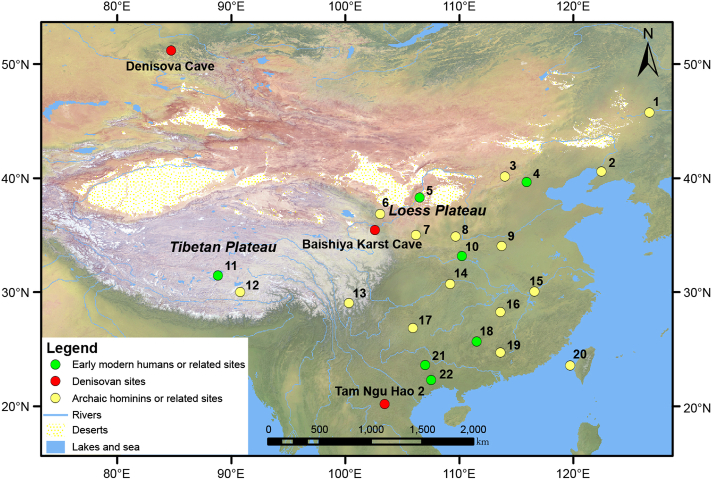
Late Middle and Late Pleistocene sites in East Asia. The numbers represent archaeological sites and/or hominin fossils as follows: 1. Harbin cranium; 2. Jinniushan; 3. Xujiayao; 4. Tianyuan cave; 5. Shuidonggou Locality 1 and 2; 6. Jiangjunfu 01; 7. Yangshang; 8. Dali; 9. Lingjing; 10. Huanglong Cave; 11. Nwya Devu; 12. Quesang; 13. Piluo; 14. Xinglong Cave; 15. Hualongdong; 16. Tongzi; 17. Guanyindong; 18. Fuyan Cave; 19. Maba; 20. Penghu 1; 21. Lunadong; 22. Zhirendong (Zhiren Cave). Detailed information is provided in Supplementary Data 1. Base maps generated by ArcGIS 10.7 using raster data from https://www.naturalearthdata.com/.

Ancient DNA analysis of several hominin fossils from Denisova Cave, Russia, has revealed the existence of a sister lineage of Neanderthals in eastern Eurasia, the so-called Denisovans^1^. On the basis of the Denisovan genetic ancestry present in several East, South and Southeast Asian populations^5,6^, it is inferred that Denisovans were widespread in eastern Eurasia during the Late Pleistocene^1^. The Xiahe mandible (named Xiahe 1) and Denisovan sedimentary mitochondrial DNA (mtDNA) from Baishiya Karst Cave (hereafter, BKC; 3,280 metres above sea level; Extended Data Fig. 1) in Ganjia Basin on the northeastern Tibetan Plateau support this assertion^3,4^. Together, they show that Denisovans occupied BKC from at least 160 thousand years ago (ka) to around 60 ka, and possibly up to around 45 ka^3,4^.

Archaeological excavations at BKC have revealed a well-preserved stratigraphy containing a rich lithic and faunal assemblage, which provides evidence of hominin occupation from at least around 190 ka to about 30 ka (ref. ^4^). However, most Pleistocene sites on the Tibetan Plateau, such as the Jiangjunfu 01 site^7^, have yielded only a few fragmentary bone specimens. Besides the small faunal assemblage from the 151 site in the Qinghai Lake Basin, which was occupied during the last deglaciation period^8^, no other zooarchaeological or palaeontological data^9^ are available for the Middle and Late Pleistocene on the Tibetan Plateau (Supplementary Information sections 1 and 2). Therefore, BKC provides a unique opportunity to study archaic hominin subsistence strategies and the faunal ecology in which it was embedded, on the high-altitude Tibetan Plateau.

**Extended Data Fig. 2:**
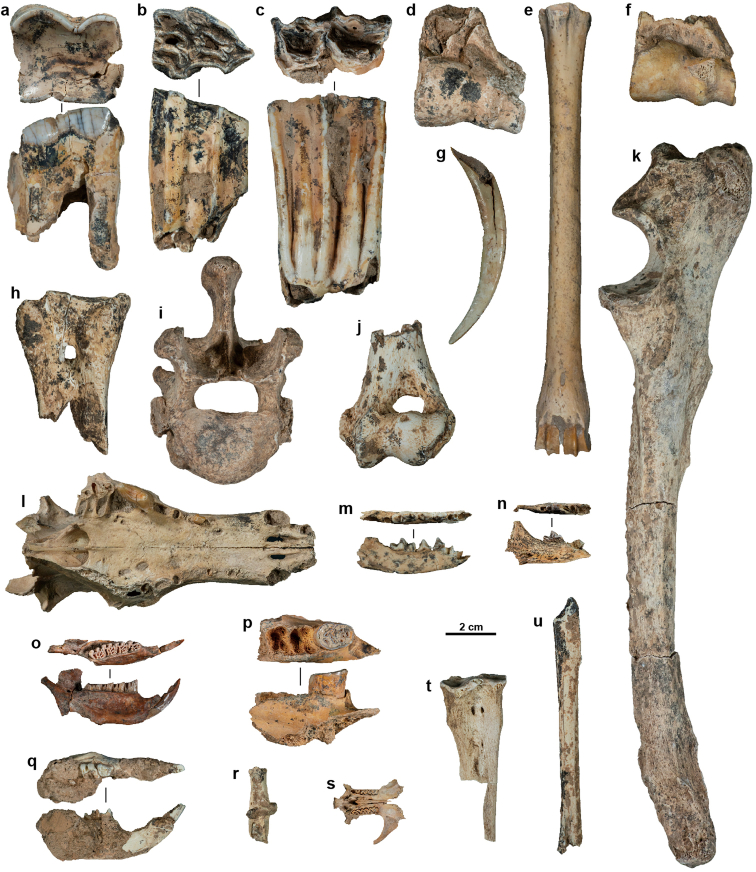
Photographs of faunal specimens recovered from BKC during the 2018 and 2019 excavations. **a**, *Coelodonta* sp. left lower M1 (layer 10b). **b**, *Equus* sp. left upper P2 (layer 10b). **c**, *Bos* cf. *mutus* left upper M3 (layer 10). **d**, *Ovis ammon* left humerus (layer 10). **e**, *Procapra* cf. *picticaudata* left metacarpus (layer 2). **f**, *Pseudois nayaur* left tibia (layer 11). **g**, *Moschu*s sp. right upper canine (layer 5). **h**, *Cervus elaphus* left metatarsal (layer 10). **i**, *Panthera* cf. *uncia* lumbar vertebra (layer 10d). **j**, *Canis lupus* left humerus (layer 11). **k**, *Crocuta crocuta ultima* left ulna (layer 10a). **l**, *Vulpes ferrilata* cranium (layer 2). **m**, *Martes* cf. *foina* left mandible (layer 11). **n**, *Mustela* sp. left mandible (layer 7). **o**, *Aeretes melanopterus* left mandible (layer 6). **p**, *Hystrix* cf. *subcristata* left mandible (layer 10a). **q**, *Marmota himalayana* left mandible (layer 10). **r**, *Lepus oiostolus* left calcaneus (layer 10c1). **s**, *Myospalax* cf. *cansus* cranium (layer 4). **t**, *Aquila chrysaetos* left tarsometatarsus (layer 11). **u**, *Phasianus* cf*. colchicus* left tibiotarsus (layer 10d). Identifications are from morphological taxonomic analysis.

We built an extended proteomic reference database of mammalian species present in or around the Tibetan Plateau through liquid chromatography with tandem mass spectrometry (LC–MS/MS) analysis (Supplementary Information section 3 and Supplementary Data 3 and 4), to allow for subsequent high-throughput zooarchaeology by mass spectrometry (ZooMS) analysis of 1,857 bone and dental specimens from BKC (Supplementary Table 2.1). Next, we integrated taxonomic identification through ZooMS with zooarchaeological data from a larger number of faunal remains (*n* = 2,567; Supplementary Table 2.1 and Extended Data Fig. 2), some of which were already taxonomically identified through morphological observations. Together, this dataset provides a novel picture of the palaeoecology and the subsistence strategies of Denisovans on the Tibetan Plateau.

## Composition of the faunal community at BKC

**Extended Data Fig. 3:**
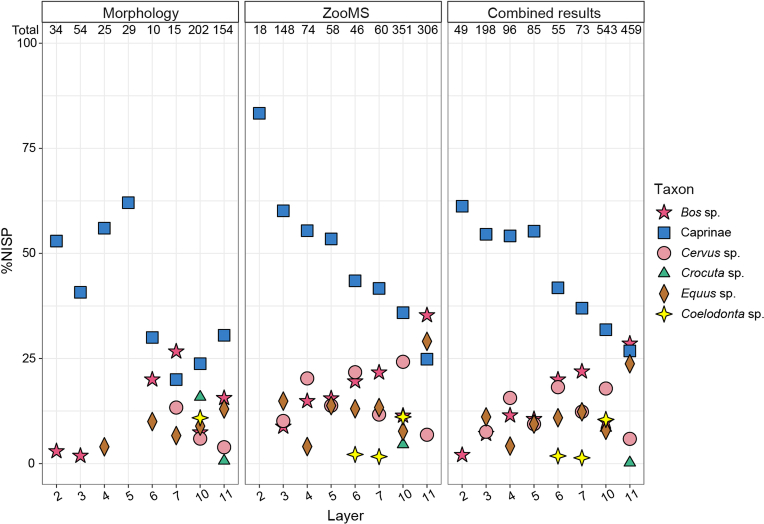
Relative species composition of BKC, for selected taxa. Proportions of selected taxa identified using morphology, ZooMS, and both combined, per layer. The total NISP for each layer is given at the top. The combined data is based on Extended Data Table 1 (*n* = 1,558).

By combining morphological and ZooMS identifications, we taxonomically identify 2,005 (78.1%) of the analysed 2,567 faunal specimens (Extended Data Fig. 2, Supplementary Data 5 and Supplementary Information section 4). Our results show that caprines (Caprinae), mostly bharal (*Pseudois nayaur*), dominate the faunal assemblage (Extended Data Fig. 3 and Supplementary Information section 4.2). The high proportion of bovids—for example, Caprinae, wild yak (*Bos* cf. *mutus*) and Tibetan gazelle (*Procapra* cf. *picticaudata*)—and equids (*Equus* sp.) throughout the stratigraphy reveals a grass-dominated landscape in the Ganjia Basin during the late Middle and Late Pleistocene (Supplementary Data 5A and Supplementary Information section 4.1). The presence of forest-shrub species, such as red deer (*Cervus elaphus*), musk deer (*Moschus* sp.), groove-toothed flying squirrel (*Aeretes melanopterus*) and porcupine (*Hystrix* cf. *subcristata*) (Supplementary Data 5A), reflects the presence of small-scale mosaic forest-shrub habitats (Supplementary Information section 4.1), similar to the modern-day foothills and river valleys in the basin^4^. In addition, various carnivores (for example, spotted hyena (*Crocuta crocuta ultima*), wolf (*Canis lupus*), Tibetan fox (*Vulpes ferrilata*) and snow leopard (*Panthera* cf. *uncia*)) and birds (for example, golden eagle (*Aquila chrysaetos*) and common pheasant (*Phasianus colchicus*)) were also present (Supplementary Data 5A).

At present, little is known about faunal community change on the Tibetan Plateau during the Middle and Late Pleistocene (Supplementary Information section 1). Notably, we only identify extinct large carnivores (*Crocuta* sp.) and megaherbivores (woolly rhinoceros (*Coelodonta* sp.)) below layer 6 (Extended Data Fig. 3 and Supplementary Fig. 4.2). Although the sample sizes for younger layers are smaller than those for layer 10 (the modelled maximum age range is around 109 ka to more than 225 ka; Supplementary Table 2.2) and layer 11 (Extended Data Table 1), the current data suggest that there was a notable change in the composition of the faunal community around BKC during the formation of layers 6 and 5 (the modelled maximum age range is around 60–104 ka, Supplementary Table 2.2). In addition, our data also document an increase in the proportion of Caprinae over time, alongside a decrease in the proportion of *Bos* sp. We cannot tell whether these changes are the result of shifts in hominin foraging strategies or more specifically related to changes in the surrounding environment (Supplementary Information section 4.3). Nevertheless, the persistent presence of bovids and equids (Supplementary Fig. 4.2) combined with small variations in taxonomic diversity (Supplementary Fig. 4.3) throughout the stratigraphy suggest a generally stable open environment in Ganjia Basin.

## Hominins were the primary accumulators

**Fig. 1:**
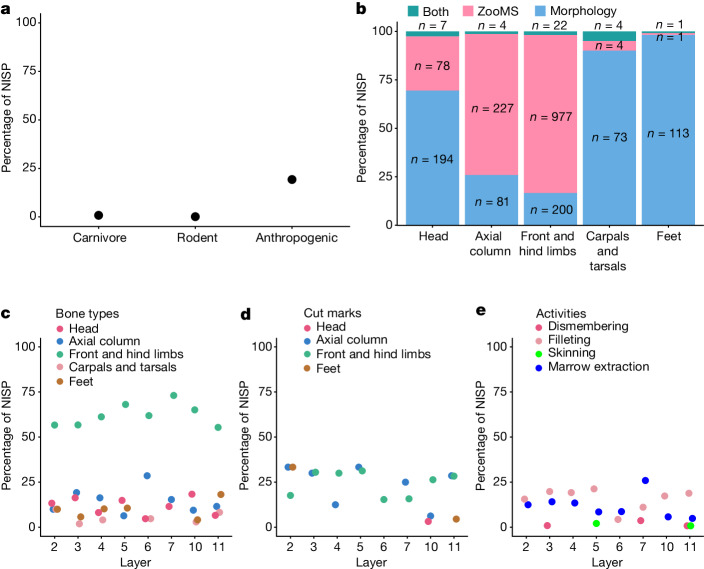
Distribution of bone surface modifications and bone types at BKC. **a**, Percentages of taxonomically identified specimens (*n* = 2,005) with carnivore, rodent or anthropogenic modifications. NISP, number of identified specimens. **b**, Percentages of taxonomically identified specimens within different bone types using morphology, ZooMS or both simultaneously (Supplementary Data 5). Bone types are based on morphological observations. The number shown on each bar is the corresponding NISP involved in the calculation. **c**, Percentages of Caprinae specimens of different bone types in each layer. **d**, Percentages of Caprinae specimens with cut marks on different bone types in each layer. **e**, Percentages of Caprinae specimens with anthropogenic modifications indicating different carcass processing activities in each layer. For **a** and **c**–**e**, values represent percentages of the NISP that fall into the respective categories.

**Extended Data Fig. 4:**
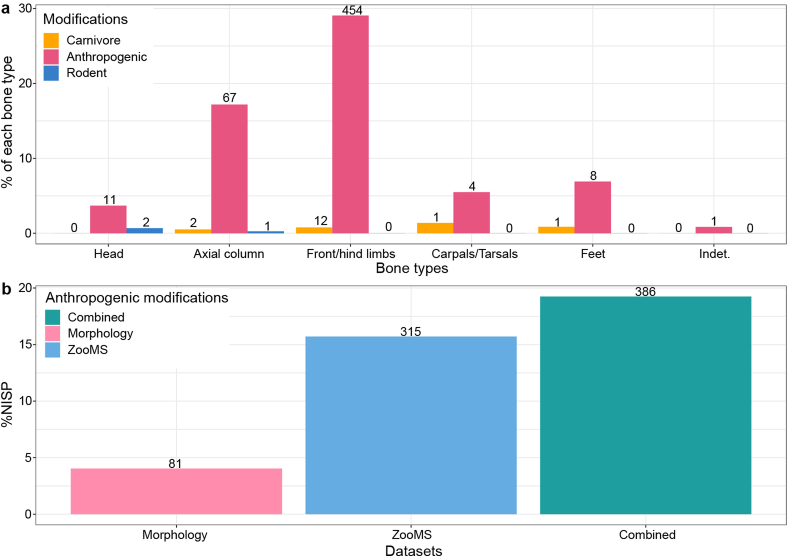
Distribution of different bone surface modifications among bone specimens. **a**, Percentages of bone specimens with different bone surface modifications among different bone types (indet. represents indeterminate specimens). **b**, Percentages of bone specimens with anthropogenic modifications identified by morphology (Supplementary Data 5A), ZooMS (Supplementary Data 5B), and both combined (Supplementary Data 5C). In **a**,**b**, the number displayed on each bar is the corresponding total NISP used in the calculation.

The surfaces of bone specimens from BKC are very well preserved, and most specimens (*n* = 1,616, 88.7% of *n* = 1,821) are within weathering stages 1 or 2, providing limited evidence for sub-aerial weathering. Traces of rodent, carnivore and anthropogenic activities were identified (Supplementary Information section 5.2 and Extended Data Fig. 4a). Rodent gnawing is limited (*n* = 3, 0.1%; Fig. 1a), with a slightly higher proportion of carnivore modified bones (*n* = 16, 0.8%; Fig. 1a), although carnivore coprolites are absent. By contrast, a larger proportion of the faunal assemblage exhibits evidence of anthropogenic modifications (*n* = 386, 19.3%; Fig. 1a). Most of these specimens were identified through ZooMS (Extended Data Fig. 4b). Cut marks and percussion notches were identified on both herbivore and some large carnivore (for example, *Crocuta* sp.) bones (Supplementary Table 5.2). The higher proportion of anthropogenically modified bones and the presence of stone artefacts in each layer^4^ suggests that the BKC faunal assemblage accumulated mainly through hominin activities (Supplementary Information section 5.2).

## Extensive anthropogenic activities

Morphologically identifiable specimens are derived largely from head fragments, carpals, tarsals and associated foot bones (Fig. 1b). ZooMS increased our taxonomic identifications and, subsequently, allowed us to successfully identify a larger number of axial and front or hind limb shaft fragments (Fig. 1b), which are usually underrepresented in skeletal profile representations^10,11^. As a result, the integration of morphologically and ZooMS identified datasets reveals a more complete composition of skeletal element structure for the whole assemblage^12–15^.

**Extended Data Fig. 5:**
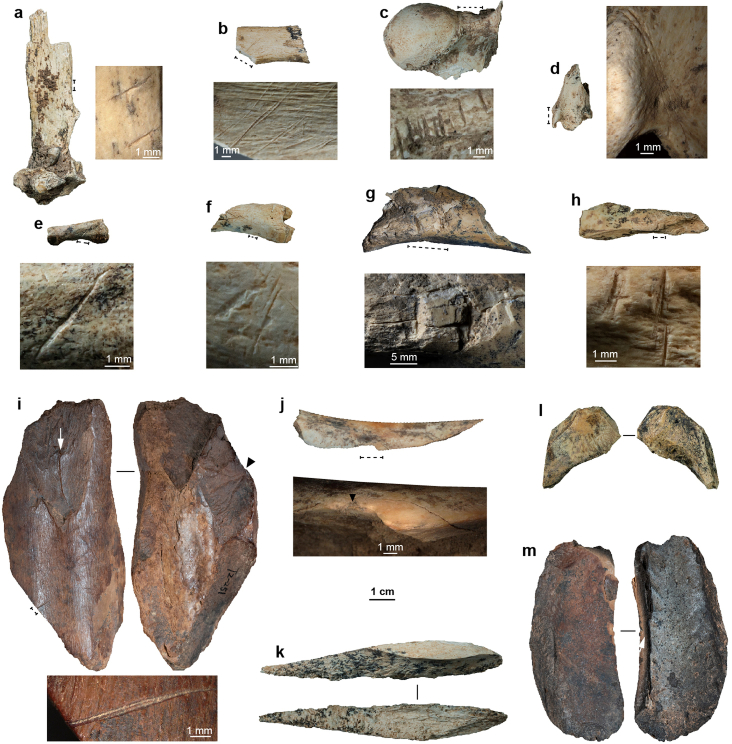
Examples of anthropogenic modifications on faunal specimens from BKC. **a**,**b**, Caprinae (layer 2) (**a**) and class II mammal (cf. Caprinae, layer 10c) (**b**) vertebral neural spines, with cut marks generated during defleshing. **c**,**d**, *Pseudois nayaur* femoral neck (layer 3) (**c**) and tibia medial malleolus (layer 11) (**d**), with cut marks created during disarticulation. **e**,**f**, *Procapra* cf. *picticaudata* (layer 10b) (**e**) and Caprinae (layer 11) (**f**) phalanx 1, with oblique cut mark(s) generated during skinning activities. **g**,**h**, Caprinae humerus diaphysis (layer 3) (**g**) and femur diaphysis (layer 11; ZooMS taxon ID) (**h**), with two parallel chop marks related to butchering activities. **i**, *Bos* sp. humerus diaphysis (layer 9), with cut marks associated with defleshing activities. Medullary edge conchoidal flaking (black triangle) and cortical percussion surface damage (white arrow) indicate the consumption of bone marrow. **j**–**l**, Caprinae radius diaphysis (layer 7) (**j**) with an adhering bone flake (black triangle), and *Bos sp*. (ZooMS taxon ID) bone flakes (layer 10a (**k**) and layer 11 (**l**)), are indications of activities of bone marrow extraction. **m**, Class III/IV mammal humerus/femur diaphysis (layer 8), with evidence of burning after the diaphysis fragment was freshly broken, and showing a sequence of colour gradients from the medullary cavity to the cortex (white arrow). Except where noted, taxonomic identifications are from morphological analysis.

In the integrated dataset (Extended Data Table 1), Caprinae are not only present in each layer, but also represented by all skeletal portions (cranial, axial, front and hind limb and foot bones) in most layers (Fig. 1c). The extent of anthropogenic activity is ubiquitous across herbivores over time, and consistently high for the Caprinae (around 20–40% for all taxonomically identified specimens; Supplementary Fig. 5.3). Cut marks related to filleting practices on Caprinae specimens are the most frequent throughout the stratigraphy, except in layers 6 and 7, in which percussion notches associated with bone marrow extraction on front and hind limb bones dominate (Fig. 1d,e, Extended Data Fig. 5 and Supplementary Fig. 5.5). Besides Caprinae, the high frequency of cut marks and percussion notches on various bone types of other herbivores, including *Bos* sp., *Cervus* sp., *Equus* sp. and *Coelodonta* sp. (Supplementary Fig. 5.3), suggests that animal resource procurement was not restricted to a particular taxon.

**Fig. 2:**
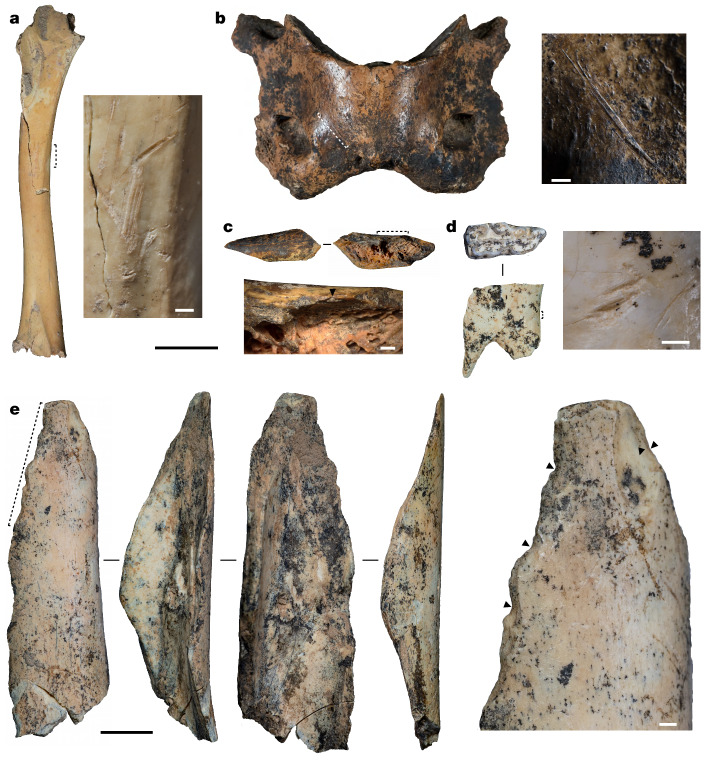
Examples of anthropogenically modified faunal specimens and bone tools. **a**, *Aquila chrysaetos* right humerus (layer 4) with superficial and straight cut mark clusters, associated with the removal of feathers. **b**, *Crocuta crocuta ultima* atlas (layer 10a), with an oblique cut mark generated during disarticulation. **c**, *Marmota* sp. (ZooMS taxon ID) radius diaphysis (layer 9), with a negative conchoidal medullary flake scar (black triangle) produced by anthropogenic breakage. **d**, A possible retoucher (layer 11). *Equus* sp. right lower P2 with a set of scrape marks on its buccal surface. **e**, Expedient bone tool (ZooMS taxon ID: Caprinae; layer 10b). This humerus diaphysis is deliberately shaped by continuous direct percussion (indicated by black triangles in the magnified image on the right) on its cortical surface. For all panels, the enlarged images (right in **a**,**b**,**d**,**e** and bottom in **c**) are magnifications of the regions denoted with dotted lines in the main images. Except where noted, taxonomic identifications are from morphological analysis. Scale bars, 2 cm (**a**–**d**, main images), 1 cm (**e**, main image) and 1 mm (all magnified images).

Anthropogenic modifications are also present on carnivores, small mammals and birds (Fig. 2 and Extended Data Fig. 5). Among the carnivore specimens (*n* = 102; Supplementary Data 5C), cut marks and percussion notches are present on several specimens (*n* = 8) from *Crocuta* sp., Pantherinae and Canidae (not *Vulpes vulpes* or *Vulpes ferrilata*) (Supplementary Table 5.2). Among the small mammals, percussion notches were observed on one radius diaphysis specimen of *Marmota* sp. (layer 9; Fig. 2c), indicating marrow extraction. In addition, seven *Lepus* sp. (layers 3, 4 and 11) and four *Marmota* sp. (layers 9–11) front and hind limb diaphysis fragments show fresh bone breakages, which are usually treated as traces of human activities. Finally, among the bird remains (*n* = 45), cut marks (*n* = 1; Fig. 2a) and fresh bone breakages (*n* = 3) are present on specimens from eagles, but not on specimens from other bird species, including pheasants, quail or the one owl specimen. Overall, zooarchaeological data suggest that the BKC hominins used a wide range of species, including large herbivores and, to a lesser extent, carnivores, small mammals and birds.

We identified one possible retoucher (Fig. 2d) from layer 11 and three expedient bone tools (Fig. 2e) from layers 4, 9 and 10 (Extended Data Table 2). The possible retoucher is produced from a tooth, and identified as *Equus* sp. Although the expedient bone tools are derived from limb bone diaphyses that have been flaked using direct percussion, ZooMS allowed us to identify these bones as belonging to Caprinae and Cervinae or *Gazella* sp. (Extended Data Table 2). Bone artefacts thus seem to have been derived from those taxa that are dominant in the BKC faunal assemblage, rather than from a deliberate focus on a single species^16^.

## A new Denisovan individual

**Fig. 3:**
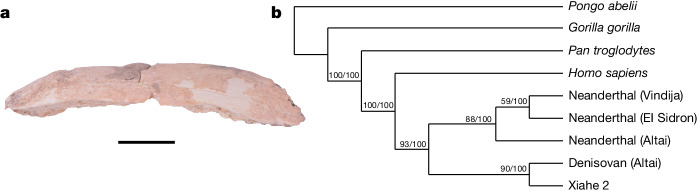
The Xiahe 2 specimen, a *Homo* sp. rib specimen discovered through ZooMS screening. **a**, Photograph of the Xiahe 2 specimen. Scale bar, 1 cm. **b**, Phylogenetic tree for the Xiahe 2 specimen and reference proteomes. Support values at nodes are shown for the maximum likelihood and Bayesian analysis, respectively.

**Extended Data Fig. 6:**
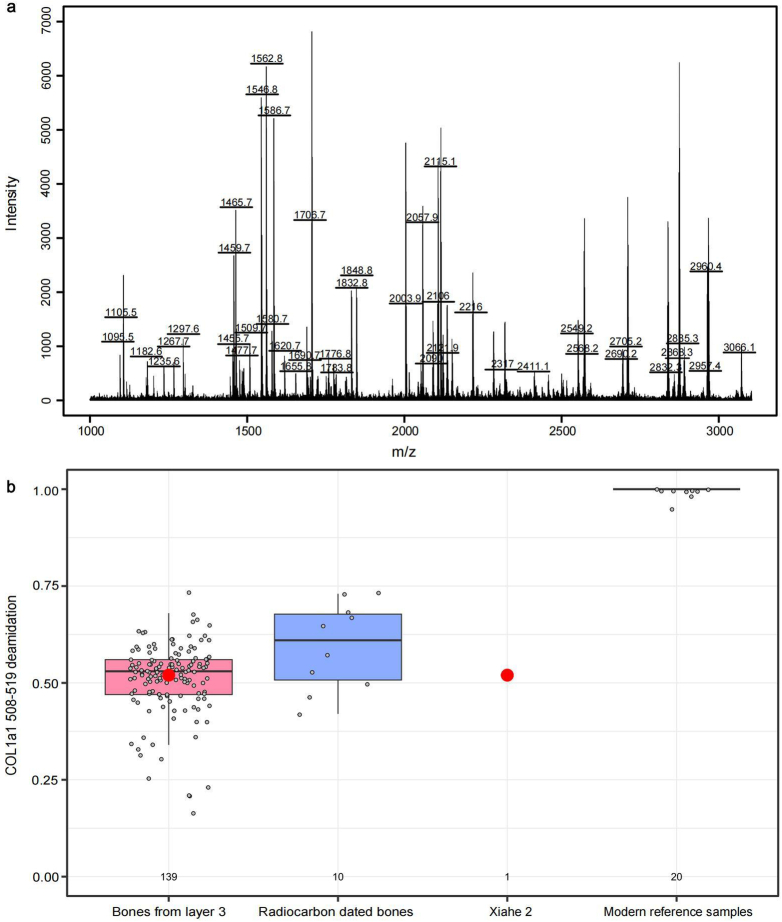
The *Homo* sp. rib specimen (Xiahe 2) identified by ZooMS. **a**, MALDI-TOF MS spectrum of Xiahe 2. **b**, Comparison of deamidation values of Xiahe 2 with values of faunal specimens from layer 3 (n = 139, including Xiahe 2, indicated by the red point), the radiocarbon-dated bone specimens (~50–30 ka, n = 10, Supplementary Data 2) and modern reference samples (n = 20). Here, the value of 1 indicates no deamidation while the value of 0 indicates complete deamidation of the single glutamine in the marker peptide COL1α1 508–519. The box plots contain the range of the data with whiskers extending to 1.5 times the interquartile range. The boxes indicate the upper and lower quartiles, while the centre line indicates the median. All individual data points are represented by overlaid dot plots, and any data points outside the range of the box plot can be considered outliers.

During ZooMS screening of the unidentifiable fragments, one rib specimen was identified as Homininae (Fig. 3a and Extended Data Fig. 6a). The specimen contains 14 peptide markers of collagen type I (COL1) matching Homininae, as well as one peptide marker unique to Hominoidea (Supplementary Data 4 and Extended Data Fig. 6a). Considering the current and past geographical distribution of other great apes^17^, in particular the genus *Pan*, this specimen could be confirmed as *Homo* sp. We therefore named this hominin specimen Xiahe 2 (field number, BSY-19-B896-1; ZooMS number, BSY-941). Xiahe 2 was broken into two pieces during excavation and belongs to the distal part of a rib (51.5 mm in length). The Xiahe 2 specimen comes from layer 3 of T3, which has been dated^4^ to 48–32 ka (Supplementary Table 2.2). The glutamine deamidation values of Xiahe 2 (acid COL1α1 508–519 = 0.52 and acid COL1α1 435–453 = 0.46) are similar to that of other specimens from layer 3 and specimens directly radiocarbon-dated to around 50–30 ka (Extended Data Fig. 6b). However, the deamidation values of Xiahe 2 are different from those of modern samples (Extended Data Fig. 6b), suggesting that the age of Xiahe 2 is consistent with the age of layer 3 (48–32 ka).

Further shotgun proteomic analyses provide more specific information about the taxonomic attribution of Xiahe 2. We reconstructed 4,597 amino acid positions for the Xiahe 2 specimen across the 21 protein sequences used for phylogenetic analysis (14.5% of the total concatenated protein sequence alignment; Supplementary Table 4.1 and Supplementary Data 6). This is a considerably larger proteome than the six endogenous proteins that were used for the phylogenetic analysis of the Xiahe 1 mandible^3^. The nodes in the phylogenetic tree have high support values through both maximum likelihood and Bayesian methods, with Xiahe 2 consistently falling together with the published high-coverage Denisovan genome (Fig. 3b). As such, it can be determined that the Xiahe 2 individual is, among the available reference individuals, most closely related to the D3 Denisovan high-coverage individual. The topology and the placement of the Xiahe 2 specimen are similar to the results obtained for the previous analysis of the Xiahe 1 mandible^3^. The discovery of the Xiahe 2 Denisovan extends the fossil evidence for the presence of Denisovans from the late Middle Pleistocene well into the Late Pleistocene at BKC, in agreement with the Denisovan sedimentary mtDNA recovered from layer 3 at BKC^4^.

## Protein preservation and deamidation

**Extended Data Fig. 7:**
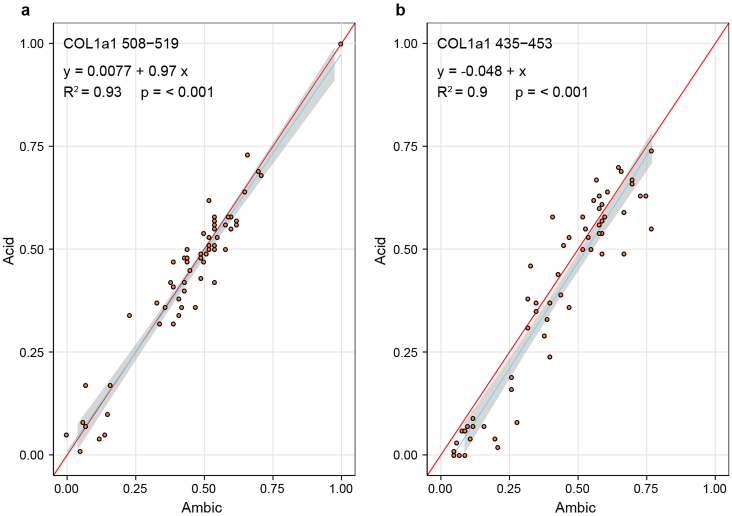
Comparison of deamidation between proteomic extraction methods. **a**,**b**, Scatter plots of COL1α1 508–519 (P1105; **a**) and COL1α1 435–453 (P1706; **b**) deamidation values for AmBic-based and acid-based extractions. The blue line and grey area indicate the mean of the loess smooth and its associated 95% confidence interval. The red line indicates a 1:1 line, where deamidation values obtained through both methods would be the same. In both cases, a high correlation is observed between the values observed for the two extraction methods (an *R*^2^ of 0.93 and 0.9, respectively).

**Extended Data Fig. 8:**
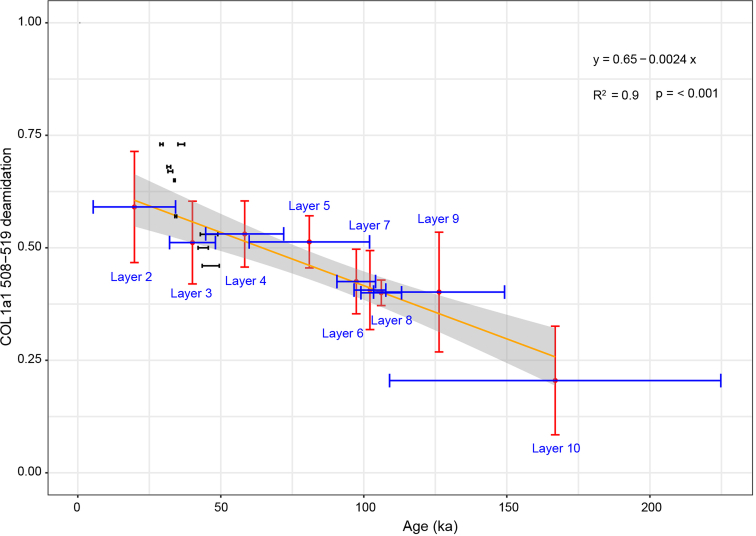
Comparison of deamidation and chronological age of the corresponding stratigraphic levels. The distribution of COL1α1 508–519 deamidation values (P1105 68.2% probability range of each layer, indicated by the red line) in relation to the chronological age (layers 2–10; black line: age ranges of specimens with direct radiocarbon dates; blue line: age ranges of each layer, see Supplementary Table 2.2). A linear correlation (orange line) is estimated across the stratigraphy (grey shadow indicates the 95% confidence interval). Layer 11 is not included here as no direct age range is currently available. In addition, layer 10 is treated as a single, homogenous stratigraphic unit (but see Supplementary Information sections 2 and 7).

Bone collagen deamidation increases gradually from top to bottom through the layers at BKC, but with considerable overlap between layers (Supplementary Information section 6 and Supplementary Fig. 6.2). The average levels of deamidation for layers 3, 4 and 5 are highly similar, as are those for layers 6 and 7. Layer 10 and, in particular, layer 11 show advanced levels of glutamine deamidation (Supplementary Fig. 6.2). Our data suggest that these results are not driven by taxonomic identity, bone length, bone type or protein extraction method (Extended Data Fig. 7 and Supplementary Fig. 6.3–6.5). However, the extent of deamidation increases gradually within the stratigraphy, in agreement with geochronological age estimates as well as with stratigraphic evidence^4^ (Extended Data Fig. 8). By contrast, the deamidation values of specimens from the historic pits, which contain a mixture of Pleistocene and Holocene remains, vary greatly. These observations are in accordance with other Pleistocene cave sites, where glutamine deamidation is generally more advanced for chronologically older and/or thermally older bone specimens in cases in which the stratigraphy spans considerable amounts of (thermal) time^18–20^.

## Discussion and conclusion

**Fig. 4:**
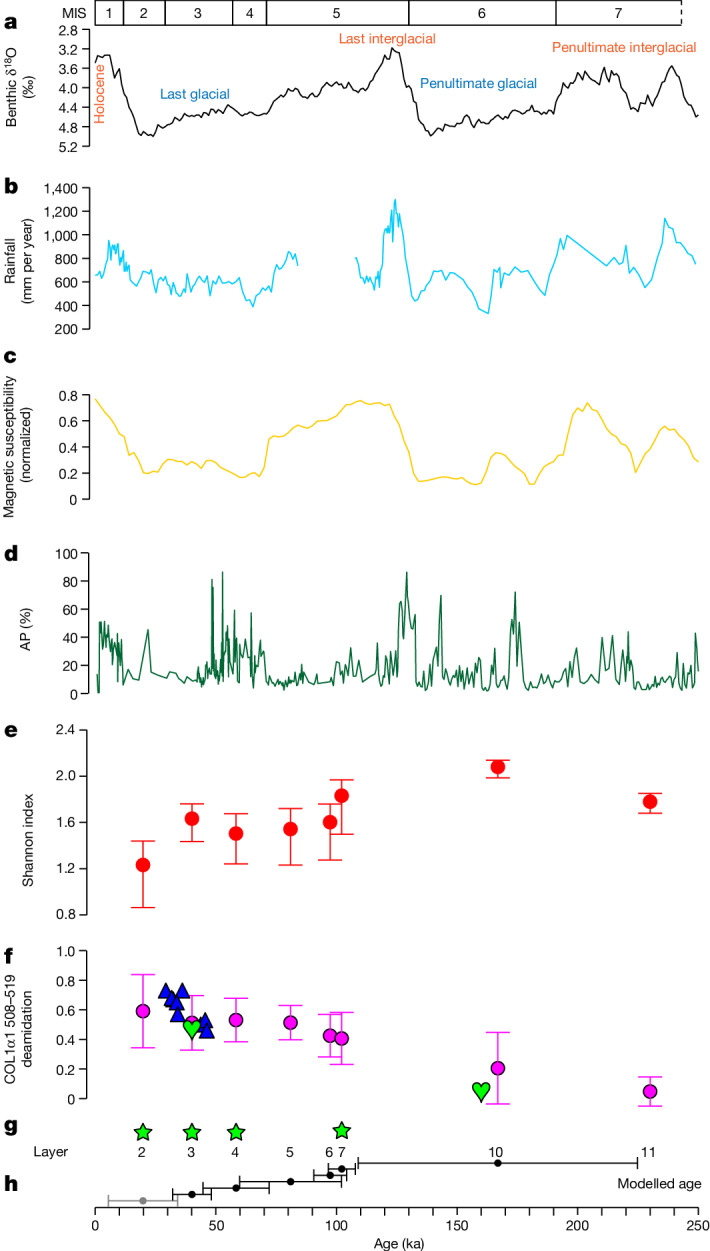
Regional and Northern Hemisphere climate history, faunal ecology and Denisovan occupation at BKC. **a**, LR04 benthic stack δ^18^O records^27^. **b**, ^10^Be-based rainfall for loess samples from Baoji, northern China^28^. **c**, Magnetic susceptibility (normalized) record from the Chinese Loess Plateau^29^. **d**, Arboreal pollen (AP) percentages (around 600-year resolution) from the Zoige Basin, eastern margin of the Tibetan Plateau^30^. **e**, Shannon index across stratigraphic units at BKC. **f**, Deamidation values for the peptide COL1α1 508–519 from bone specimens. The purple points and error bars represent the mean deamidation values of bones from each layer with 68.2% probability ranges. The blue triangles represent the deamidation values of individual bone specimens from layers 2–3 with radiocarbon dates (*n* = 9, Supplementary Data 2). The green hearts represent the deamidation values of the two Denisovan specimens (Xiahe 1 and 2). **g**, Stratigraphic layers and Denisovan sedimentary mtDNA. Denisovan mtDNA extracted from sediments in layers 2, 3, 4 and 7 is indicated by green stars. **h**, Modelled age range of each layer. The black points and the bar ranges indicate the modelled mean age and age range for each layer (Supplementary Table 2.2). The age range of layer 2 is still under evaluation, and there are currently no dates for layers 5 and 11. The indicated age range estimate for layer 5 is based on the age interval between layers 4 and 6. Detailed chronological information is available in Supplementary Information section 2.

Previous studies show that BKC is currently the only well-preserved cave site on the Tibetan Plateau that spans the late Middle to Late Pleistocene^3,4^ (Supplementary Information section 1). The Xiahe 1 mandible and sedimentary mtDNA analyses reveal that Denisovans occupied the cave at least around 160 ka, 100 ka (layer 7) and 60 ka (layer 4), and possibly as late as 45 ka (end layer 4)^3,4^. The Xiahe 2 rib identified here, and Denisovan sedimentary mtDNA discovered from layer 3 (ref. ^4^), show that Denisovan occupation occurred at the site until at least 48–32 ka. Layers 10 and 11 at BKC provide the richest archaeological remains in the cave, including more than 60% of the bone specimens analysed here (Supplementary Table 2.1), but unfortunately without any hominin remains or sedimentary mtDNA to ascertain the biological identity of the occupants so far. However, the deamidation values obtained for Xiahe 1, COL1α1 508–519 (0.04) and COL1α1 435–453 (0.00) are, exclusively, within the range of those observed for bone specimens from layers 10 and 11 (Fig. 4f). In addition, Xiahe 1 has a minimum U-series age of around 160 ka (ref. ^3^), which also corresponds to the chronological age of layer 10 or below (Fig. 4g). Although this does not show that Xiahe 1 definitely derives from layers 10, 11 or an older layer at BKC, it does show that the Xiahe Denisovans were the most likely occupants during the formation of these layers.

So far, there is no evidence for the presence of other hominins at BKC for layers 3–11, nor is there evidence for other archaic hominin occupation elsewhere on the northeastern Tibetan Plateau during the same period (Supplementary Information section 1). It is therefore reasonable to assume that Denisovans occupied BKC at least from around 167 ka (modelled mean age for layer 10) to around 40 ka (modelled mean age for layer 3), and possibly from more than 224 ka (modelled maximum age for layer 10) to 32 ka (modelled minimum age for layer 3) (Supplementary Table 2.2). Thus, the BKC faunal assemblage documents Denisovan behaviour and subsistence in the Ganjia Basin during the last glacial–interglacial–glacial cycle: the penultimate glacial period, marine isotope stage (MIS) 6, represented by the lower sublayers of layer 10 and possibly layer 11; the last interglacial period, MIS 5e, represented by the transition layer 10/9 (149.2–109 ka) and possibly layer 10a; and the last glacial period, MIS 4 and 3, represented by layers 4 and 3 (Fig. 4).

The comprehensive analysis of the faunal assemblage from BKC shows that Denisovans exploited a wide range of animal taxa that were present in the grass-dominated landscape around Ganjia Basin. The role of Caprinae becomes increasingly prominent during hominin occupation, especially in layers 5 to 2, after MIS 5, in which Caprinae specimens compose over half of the faunal assemblage (Extended Data Fig. 3). Analyses of anthropogenic modifications on Caprinae specimens indicate that the complete *chaîne opératoire* of carcass processing, including systematic butchery, and the use of bone material for tools, is present at BKC (Supplementary Information section 5.3). In addition to Caprinae, the remains of megaherbivores, carnivores, small mammals and birds were similarly used in a variety of ways. This reveals that Denisovans made full use of the animal resources available to them in order to survive on the high-altitude Tibetan Plateau during the last glacial–interglacial–glacial cycle. During both glacial and interglacial periods, the Ganjia Basin might have provided a suitable refugium with relatively stable resource availability despite its altitude, especially in comparison with higher-altitude regions of the Tibetan Plateau or the fluctuating environmental conditions on the neighbouring Chinese Loess Plateau^21–23^.

By comparing BKC with other Denisovan or possible Denisovan sites, namely Denisova Cave in Russia and Tam Ngu Hao 2 (Cobra) Cave in Laos, we find that their faunal assemblages are compatible with their respective geographical environments, corresponding to high-altitude, high-latitude and tropical (or subtropical) environments (Supplementary Information section 4.1). Our results therefore provide evidence for both palaeoecological and behavioural plasticity in Denisovans. Furthermore, these insights raise questions as to the cause and timing of Denisovan extinction on the Tibetan Plateau, as well as the origins of Denisovan genetic signatures in modern humans^6,24–26^.

## Methods

### Baishiya Karst Cave

Baishiya Karst Cave (BKC; 35.45° N, 102.57° E, 3,280 metres above sea level) is located in the Ganjia Basin, northeastern Tibetan Plateau^4^ (Extended Data Fig. 1 and Supplementary Information section 2). It is a karstic cave and lies around 20 m above the riverbed of the Jianglagou river in front of the cave. The Xiahe mandible (Xiahe 1) was found in this cave in 1980 and has been dated to at least 160 ka by U-series dating of carbonate crust on the mandible^3^. The Xiahe 1 individual has been identified as a Denisovan by palaeoproteomic analysis^3^. Two connected units (T2 and T3, 1 m × 2 m, respectively) were excavated in this cave in 2018 and 2019, revealing 11 layers. The chronological framework for layers 2–10 built by optically stimulated luminescence (OSL) and radiocarbon dating methods indicates that prehistoric hominins occupied the cave from around 190 ka to around 30 ka (ref. ^4^). Denisovan mtDNA extracted from sediments of layers 4 and 7 indicates that the site was occupied by Denisovans at around 100 ka, around 60 ka and possibly as late as 45 ka (ref. ^4^), providing further unequivocal evidence of Denisovan occupation at BKC. In addition, Denisovan mtDNA was also recovered from layers 2 and 3, and the age of this is still under detailed calculation and evaluation.

### Chronological framework

A previous study^4^ used OSL and radiocarbon dating to establish a chronological framework for layers 2–10 of T2. In this study, we apply this framework to both T2 and T3, but use the maximum age range to represent the age of each layer (Supplementary Table 2.2). See Supplementary Information section 2 for details.

### Sample selection

A total of 3,642 bone specimens were systematically collected from T2 and T3 during excavations in 2018 and 2019, 3,582 of which were recorded with three-dimensional coordinates, all stored in the Key Laboratory of Western China’s Environmental Systems (Ministry of Education) at Lanzhou University. Among these, we selected almost all bones longer than 20 mm (*n* = 2,407), as well as some smaller bone fragments (shorter than 20 mm) with morphological characteristics (*n* = 160) suitable for morphological taxonomic analysis, for a total of 2,567 specimens (Supplementary Table 2.1). Among these specimens, 2,407 specimens were uncovered from layers 1 to 11, 138 specimens derived from two historical-era pits (H1 and H3) and 22 specimens lacked specific layer information. Because layers 1–10 are seriously destroyed by the two historical pits (H1 and H3), only parts of these layers are preserved^4^, resulting in the small number of bones (*n* = 764) collected from layers 1–9. Therefore, about 64% of the analysed bone assemblage derives from layers 10 and 11 (*n* = 1,643). In this assemblage, we selected 1,857 specimens for ZooMS analysis, including 53 specimens with taxonomic identifications based on morphological analysis for proteomic confirmation of their taxonomic assignments (Supplementary Table 2.1).

### ZooMS reference samples

Current ZooMS reference databases are dominated by fauna from western Eurasia, supplemented with family-specific reference datasets. In addition, a variety of predicted peptide mass marker series from COL1 sequences exist based on available genomic resources. Nevertheless, a substantial number of mammalian taxa that are potentially present at BKC are not represented in any of these resources. We therefore collected 39 specimens with known taxonomic information, representing 39 species and 29 genera, that are known to currently inhabit the wider Himalayan region, or are known to have been present in this region in the Pleistocene or Holocene (Supplementary Data 3). Among these reference specimens, ten are of Pleistocene or Holocene age, whereas others are modern specimens. The 39 reference specimens also included 6 specimens from species for which genomic sequences are available but for which no reference matrix-assisted laser desorption ionization time-of-flight mass spectrometry (MALDI-TOF MS) collagen type I peptide mass fingerprints have been reported before, such as the giant panda (*Ailuropoda melanoleuca*) and the Tibetan antelope (*Pantholops hodgsonii*). Reference specimens were collected from Lanzhou University, the Institute of Zoology, Chinese Academy of Sciences (CAS) and the Northwest Institute of Plateau Biology, CAS (Supplementary Data 3).

After obtaining peptide mass fingerprints for each reference specimen (see extraction method below), 14 species, representing 14 genera, were selected for LC–MS/MS analysis to validate (novel) peptide marker masses.

### Zooarchaeological analysis

Diagnostic skeletal elements were identified using modern and ancient comparative vertebrate specimens from the Key Laboratory of Western China’s Environmental Systems (Ministry of Education) at Lanzhou University and comparative osteological atlases^31–37^. The identified taxa were classified into six body-size classes (class 0–class V) based on live weight, following previously established criteria^38^: class I (*Marmota himalayana*, *Lepus oiostolus*, *Hystrix* sp., Mustelidae, *Vulpes ferrilata*); class II (*Procapra* sp., *Moschus* sp., *Panthera* sp., *Canis lupus*); class III (*Pseudois nayaur*, *Ovis ammon*, *Cervus elaphus*, *Crocuta crocuta*); class IV (*Equus* sp., *Bos mutus*); and class V (*Coelodonta* sp.). For carnivores, large animals correspond to class II or class III, and small animals correspond to class I. For herbivores, mega, large, medium and small animals correspond to classes V, IV, III or II and I, respectively. In addition, birds are classified as class 0.

Each specimen was observed under a portable low-magnification hand lens (20×) using an oblique light source, and was observed and photographed using a Keyence VHX-7000N digital microscope at different magnifications when necessary. Bone surface modifications, including anthropogenic modifications (cut and chop marks, percussion marks and notches and impact bone flakes), animal gnawing traces (pits, punctures, scores, furrowing and so on), trampling and modern mechanical modifications produced by excavation tools, were recorded according to the literature^39–43^. The weathering stages of the bone surface were classified into four levels (I–IV) according to the classical criteria^39^. We also recorded burnt bone specimens on the basis of macroscopic colour changes during exposure to fire^44^. The fracture types of bone fragments longer than 20 mm were recorded and classified according to previously described criteria^45^. The five anatomical regions in this paper are head (including horn and antler); axial column (including vertebrae, ribs and pelvis); front and hind limbs (including scapulae, humeri, radii and ulnae, metacarpi, femurs, tibiae and metatarsals); carpals and tarsals; and feet, on the basis of the structure defined in a previous report^46^.

Bone retouchers were identified according to previously described criteria^47^. Expedient bone tools were identified on the basis of quantitative criteria described previously^48,49^. Specifically, bone fragment specimens with more than four to six flake scars resulting from knapping and/or retouching, arranged with a high frequency of continuity and/or interspersion, can be interpreted as having been formed by purposeful percussion and are identified as expedient bone tools.

For our study, measures of taxonomic abundance are based on the number of identified specimens (NISP) rather than the minimum number of individuals (MNI). On one hand, the NISP can be compared quantitatively with the ZooMS data, which are essentially a NISP count. On the other hand, the MNI of most taxa in each layer is 1 or 2 (Supplementary Data 5A), which is not conductive to estimates of taxonomic abundance. The NISP in our study is calculated as the number of specimens identified to species (for example, *Aquila chrysaetos*) and genus (for example, *Coelodonta* sp.), and occasionally to (sub)family (for example, Caprinae)^50^.

### ZooMS collagen extraction and digestion

We sampled approximately 10–30 mg of each specimen. Two protocols were applied to the BKC bone samples as well as the reference samples: the non-destructive ammonium bicarbonate buffer (AmBic) extraction method^51^ and the acid-insoluble (acid) extraction method^19^. Details for both protocols have been provided previously^12,19^. The acid protocol was applied to all reference samples and all BKC specimens. In addition, the AmBic protocol was applied to all reference samples and 192 BKC specimens. In brief, before extraction, all of the bone samples were stored in 100 µl AmBic solution overnight, to remove any soluble contamination. After removing this solution, for the AmBic protocol, 100 µl ammonium bicarbonate (50 mM) was added to the bone samples and followed by incubation for one hour at 65 °C. Then, 50 µl of the supernatant containing the soluble protein was transferred to a new 96-well plate or Eppendorf tube and digested with trypsin (Promega, V115A) at 37 °C overnight. Digestion was terminated with 1 µl of 5% trifluoroacetic acid (TFA). Peptide clean-up and purification was done on C18 ZipTips (Thermo Fisher Scientific) or C18 plates (Thermo Fisher Scientific), with elution in 50 µl or 100 µl, respectively, with 0.1% TFA washing solution and 0.1% TFA in 50% acetonitrile as conditioning and elution solution. For the acid protocol, bone samples were demineralized in 0.6 M hydrochloric acid (HCl) solution for one to two days. The HCl was discarded, and the pellet was washed three times with 50 mM AmBic until the pH was around 8. Subsequent steps were identical to the AmBic protocol. All reference samples were processed in individual Eppendorf tubes, and the BKC bone samples were processed using 96-well plates.

### Extraction of the Xiahe 2 bone proteome

Four proteomic digests were generated and used for LC–MS/MS analysis of the Xiahe 2 rib specimen, deriving from a total of two bone samples taken from this specimen. Both samples were first stored overnight in 50 mM AmBic to remove any soluble protein contamination potentially present on the bone surfaces. Subsequently, the first protein extraction of the first sample concerned an AmBic ZooMS extraction in which the sample was incubated in 50 mM AmBic for one hour at 65 °C. Digestion of the solubilized proteins within the resulting supernatant was subsequently performed in solution and overnight, at 37 °C (trypsin, Promega, V115A). The remaining pellet as well as the second bone sample were subsequently demineralized in 0.6 M HCl for one or two days, until demineralization was observed. The extracts were centrifuged, and the acidic solution containing acid-soluble proteins was removed, dried down in a speedvac and resuspended in 50 mM AmBic. Subsequently, protein digestion was performed overnight using trypsin at 37 °C. Finally, the fourth extract concerned the resuspension of the remaining, demineralized protein pellet of the first sample in 50 mM AmBic. Again, protein digestion was conducted using trypsin at 37 °C. In each case, peptides were acidified to terminate the digestion process using 1% TFA, briefly centrifuged to pellet any remaining mineral or protein residues and purified using C18 ZipTips as described above for ZooMS. LC–MS/MS analysis was performed on 10 µl of each of the four peptide eluates.

### MALDI-TOF MS

For MALDI-TOF MS analyses, 1 µl of the eluted peptides was mixed with 1 µl of matrix solution (1% α-cyano-4-hydroxycinnamic acid in the conditioning solution) and spotted onto a 384-well MALDI target plate. Each sample was spotted in triplicate. All of the MS spectra data were obtained at the Fraunhofer IZI, Leipzig, using the Autoflex Speed LRF MALDI-TOF (Bruker). Spectral replicate merging was performed in R using the MALDIquant v.1.22.1 and MALDIquantForeign v.0.14 packages^52–54^. The peptide marker masses were observed in mMass^55^, and identified in comparison with the updated ZooMS database (Supplementary Data 4).

### LC–MS/MS analysis

Ten microlitres of the eluted peptides of 14 species (Supplementary Data 3) was processed using LC–MS/MS analysis at the University of Copenhagen.

For liquid chromatography, an easy NanoLC from Thermo Fisher Scientific, with the gradient specified in Supplementary Table 3.1, was used at 250 nl per min. The loading was made at 500 nl per min. The mobile phases are A: 5% acetonitrile, 0.1% formic acid; B: 95% acetonitrile, 0.1% formic acid. The emitter consisted of a Polymicro flexible fused silica capillary tubing of 75 µm inner diameter and 20 cm length home pulled and packed with C18 bounded silica particles of 1.9 µm diameter (ReproSil-Pur, C18-AQ, Dr. Maisch). The column was mounted on an electrospray source with a column oven set at 40 °C.

For mass spectrometry, the source voltage was +2,000 V with an ion transfer tube set at 275 °C. An Exploris 480 from Thermo Fisher Scientific was operating in data-dependent mode consisting of a first MS1 scan at a resolution of 120,000 between *m*/*z* values of 350 and 1,400. The top ten monoisotopic precursors were selected if above an intensity of 2 × 10^4^ with a charge state between 2 and 6, and were then dynamically excluded after one appearance with their isotopes (±20 ppm) for 20 s. The selected peptides were acquired for MS2 at an Orbitrap resolving power of 60,000, with a normalized collision energy (HCD) set at 30%, a quadrupole isolation width of 1.2 *m*/*z* and a first *m*/*z* of 100.

### COL1 peptide marker validation

Peptide sequences were acquired from the LC–MS/MS raw data using PEAKS v.7.0 (ref. ^56^), with closely related species with available collagen sequences forming reference databases. Deamidation (NQ), hydroxylation (P) and oxidation (M) were set as variable modifications, and no fixed modifications were included. Parent mass error tolerance was set to 10 ppm, fragment ion tolerance to 0.07 Da and trypsin as the protease. Peptides were filtered for a false discovery rate (FDR) of 0.5%, on the basis of previous conservative recommendations made for PEAKS analysis of skeletal palaeoproteomes^57^, and sequence reconstruction was performed in R^52^ using the packages Tidyverse v.2.0.0 (ref. ^58^), Janitor v.2.2.0 (ref. ^59^), Biostrings v.2.68.1 (ref. ^60^) and msa^61^. Polymorphisms between the references and the reconstructed sequence within the peptide marker were manually validated in PEAKS.

### LC–MS/MS analysis of Xiahe 2

Peptide sequences were identified from LC–MS/MS data using PEAKS v.7.0 (ref. ^56^) with a database consisting of the human reference proteome (UP000005640 with one protein sequence per gene, downloaded 22-02-2022) with added archaic variation from Neanderthals^62^ and a Denisovan^63^. Parent mass error tolerance was set to 10.0 ppm and fragment mass error tolerance to 0.07 Da. Deamidation (NQ), hydroxylation (P), oxidation (M) and pyro-Glu (E and Q) were set as variable modifications. Peptides were filtered for 0.5% FDR and exported for further processing.

### Construction of the Xiahe 2 protein sequences and phylogenetic analysis

For proteomic data from the Xiahe 2 individual, protein sequences were reconstructed for all proteins with five or more peptides (Supplementary Table 4.1 and Supplementary Data 6) in R^52^ using the packages Janitor v.2.2.0 (ref. ^59^), Biostrings v.2.68.1 (ref. ^60^) and Tidyverse v.2.0.0 (ref. ^58^). The proteins.csv file exported from PEAKS was used to get an overview of the proteins present in the dataset and their abundance, whereas the protein-peptides.csv file was used for sequence reconstruction. For each amino acid position, a majority consensus was called on the basis of peptide counts. The reconstructed sequences were thereafter aligned using Geneious Prime v.2023.2.1 with a list of reference proteomes, and all polymorphisms were manually validated in PEAKS. The reference proteomes used were the human reference proteome UP000005640 (downloaded from Uniprot 17-01-2022), translations of three Neanderthal genomes^62^ and a translation of a Denisovan genome^63^.

A phylogeny was constructed using the reference proteomes outlined above, as well as corresponding proteins from *Gorilla gorilla*, *Pongo abelii* and *Pan troglodytes*. Sequences were aligned in Geneious Prime to identify and correct for isoform variations between references. In brief, positions 1264–1270 (based on P29400) of COL4A5 were removed for the Neanderthals and *P. troglodytes*; positions 261–313 (based on P12107) of COL11A1 were removed for all individuals; 235 unknowns were added after position 219 (based on P39060) of COL18A1 for *G. gorilla*, owing to missing portions of the reference sequence; and positions 3–21 of COL27A1 (based on Q8IZC6) for *G. gorilla* were replaced with unknowns owing to major sequence differences. Protein sequences were concatenated by the individual, with a concatenated protein length of 31,781 amino acid positions.

Phylogenetic analyses were performed using the Dayhoff substitution model and partitioning by protein. The *P. abelii* sequence was used as an outgroup. A Bayesian tree was generated using MrBayes v.3.2.7 (ref. ^64^), with settings following a previous report^3^, except for the analysis only being run for 100,000 generations, because the standard deviation of split frequencies was already zero by this point. RAxML v.4.0 (ref. ^65^) analysis was run through Geneious Prime, with 1,000 bootstraps (rapid bootstrapping with search for best-scoring ML tree). The phylogeny was plotted in R using Ape v.5.7.1 (ref. ^66^).

### Faunal community analysis

To better compare datasets obtained by morphological observations and ZooMS, we united all results under ZooMS taxonomic group levels, because these are generally less specific than the species designations obtained through morphological analysis. For example, we assign the taxa *Bos* cf. *mutus* and *Bos grunniens* from the morphological taxonomic groups into *Bos* sp., because these species cannot be separated using ZooMS. However, to better display the morphological data, we kept the species information of some specimens by using the species names, such as *Bos grunniens* or *Bos* cf. *mutus*, in some places when necessary. Taxon names used in the text are according to the specific datasets (morphology, ZooMS and both combined) and the source dataset information is indicated wherever necessary. The combined dataset, a hybrid of morphological and ZooMS taxonomic identifications, was used to analyse the composition of the faunal community at BKC and to calculate diversity indices. Within this dataset, we excluded groups such as birds, rodents and taxonomic assignments to inter-order or sub-order levels.

The Shannon–Weaver and Simpson indices, including the confidence intervals (97.5–2.5%), were calculated according to a previously described method^67^ to assess the community ecology of each stratigraphic unit. These were calculated in R^52^ using the vegan v.2.6-4 package^68^. The values of Shannon–Weaver and Simpson indices are positively proportional to diversity, suggesting that higher index values indicate higher diversity.

### Deamidation

The deamidation of glutamine (Gln; Q) to glutamic acid (Glu; E) in bone specimens is one of the common post-translational modifications in archaeological contexts. According to previous studies^69,70^, an effective method has been proposed to calculate glutamine deamidation for selected collagen type I peptides. Here we calculate glutamine deamidation for two peptides, COL1α1 508–519 (GVQGPPGPAGPR; P1105) and COL1α1 435–453 (DGEAGAQGPPGPAGPAGER; P1706), that have relatively slow deamidation rates^69^. They are also commonly present in BKC MALDI-TOF MS spectra, and their peptide sequences are identical for most terrestrial mammals^19^. Deamidation is expressed on a scale of 0 to 1, with 1 indicating no deamidation and 0 indicating complete deamidation.

### Reporting summary

Further information on research design is available in the Nature Portfolio Reporting Summary linked to this article.

## Online content

Any methods, additional references, Nature Portfolio reporting summaries, source data, extended data, supplementary information, acknowledgements, peer review information; details of author contributions and competing interests; and statements of data and code availability are available at 10.1038/s41586-024-07612-9.

### Supplementary information

Supplementary Information This file contains Supplementary Information sections 1–7, including 16 Supplementary Figures and 8 Supplementary Tables, and references

Reporting Summary

Supplementary Data 1 A list of Late Middle and Late Pleistocene sites in East Asia

Supplementary Data 2 Radiocarbon and deamidation results of bones from T2.

Supplementary Data 3 Collected reference samples for MALDI-TOF MS and LC–MS/MS analyses.

Supplementary Data 4 An extended database of ZooMS peptide marker masses and peptide marker sequences.

Supplementary Data 5 The taxonomic assignments per layer. A. Morphological taxonomic identifications. B. ZooMS taxonomic identifications. C. Taxonomic identifications after combining morphological and ZooMS taxonomic identifications.

Supplementary Data 6 Consensus sequences derived for the Xiahe 2 specimen after shotgun proteomic analysis, and used for phylogenetic analysis.

## Figures and Tables

**Extended Data Table 1:**
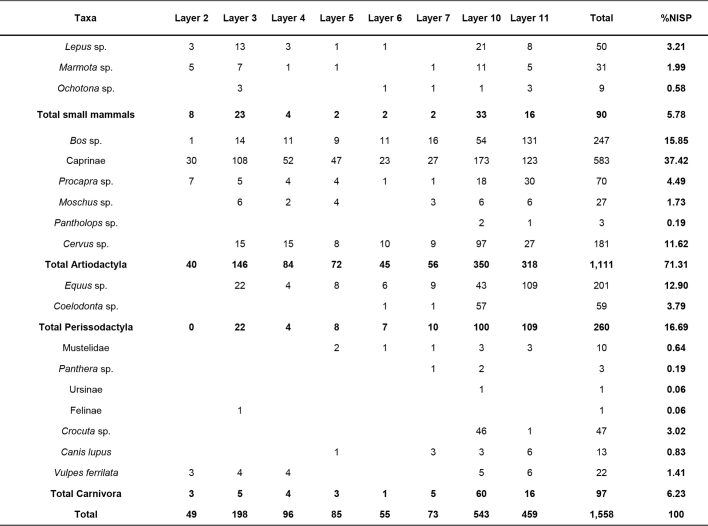
Distribution of mammalian taxonomic groups after combining the morphological and ZooMS taxonomic identifications, across stratigraphic units

This table only includes clearly classified mammalian taxonomic groups from layers 2–7 and 10–11 (*n* = 1,558). See Supplementary Data 5A,B for tabular data on separate morphological and ZooMS taxonomic compositions, and Supplementary Data 5C for the combined dataset and all taxonomic groups from all stratigraphical units (*n* = 2,005).

**Extended Data Table 2:**
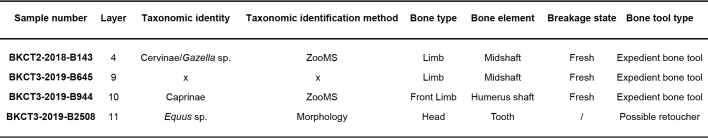
Overview of the three expedient bone tools and a possible tooth retoucher recovered from BKC

For the taxonomic identity column, these refer to ZooMS or morphological taxonomic assignments, with ‘x’ representing a specimen that did not receive a taxonomic identification through either approach.

## Data Availability

The mass spectrometry proteomics data have been deposited to the ProteomeXchange Consortium via the PRIDE^71^ partner repository with the dataset identifiers PXD041874 and PXD047932.

## Code availability

All R code used for protein sequence reconstruction is available through Zenodo at 10.5281/zenodo.11281297 (ref. ^72^).
