# Supplementary material for: Middle and Late Pleistocene Denisovan subsistence at Baishiya Karst Cave
Source: Nature. 2024 Jul 3;632(8023):108–13. doi: 10.1038/s41586-024-07612-9 (PMC11291277; doi:10.1038/s41586-024-07612-9)
Supplement: Supplementary file 1 — This file contains Supplementary Information sections 1–7, including 16 Supplementary Figures and 8 Supplementary Tables, and references [file 41586_2024_7612_MOESM1_ESM.pdf]

nature portfolio
https://doi.org/10.1038/s41586-024-07612-9

Supplementary information

Middle and Late Pleistocene Denisovan subsistence at Baishiya Karst Cave

In the format provided by the authors and unedited

Supplementary information to:

Middle and Late Pleistocene Denisovan subsistence at Baishiya Karst Cave

Huan Xia[1,2,3,13], Dongju Zhang[1,2,13]*, Jian Wang[1,4,13], Zandra Fagernäs[5,13], Ting Li[1], Yuanxin Li[1], Juanting Yao[1], Dongpeng Lin[1], Gaudry Troché[5], Geoff M. Smith[6,7], Xiaoshan Chen[1], Ting Cheng[1], Xuke Shen[1], Yuanyuan Han[1,2], Jesper V. Olsen[8], Zhongwei Shen[1], Zhiqi Pei[1,9], Jean-Jacques Hublin[10,11], Fahu Chen[2,12,1]*, Frido Welker[5]*

1. Key Laboratory of Western China's Environmental Systems (Ministry of Education), Key Scientific Research Base of Bioarchaeology in Cold and Arid Regions (National Cultural Heritage Administration), College of Earth and Environmental Sciences, Lanzhou University, Lanzhou, China.
2. Alpine Paleoecology and Human Adaptation Group (ALPHA), State Key Laboratory of Tibetan Plateau Earth System, Environment and Resources (TPESER), Institute of Tibetan Plateau Research (ITPCAS), Chinese Academy of Sciences (CAS), Beijing, China.
3. College of Ecology, Lanzhou University, Lanzhou, China.
4. School of Earth Sciences, Lanzhou University, Lanzhou, China.
5. Globe Institute, University of Copenhagen, Copenhagen, Denmark.
6. School of Anthropology and Conservation, University of Kent, Canterbury, United Kingdom.
7. Department of Archaeology, University of Reading, Whiteknights Campus, Reading, UK.
8. Novo Nordisk Foundation Center for Protein Research, University of Copenhagen, Copenhagen, Denmark.
9. Gansu Provincial Museum, Lanzhou, China.
10. Chaire de Paléoanthropologie, CIRB, Collège de France, Université PSL, CNRS, Paris, France.
11. Max Planck Institute for Evolutionary Anthropology, Leipzig, Germany.
12. University of Chinese Academy of Sciences, Beijing, China.
13. These authors contributed equally: Huan Xia, Dongju Zhang, Jian Wang, Zandra Fagernäs.

* Corresponding authors. Email: frido.welker@sund.ku.dk; djzhang@lzu.edu.cn; fhchen@itpcas.ac.cn.

This PDF file includes:
Supplementary information

Section 1: Middle and Late Pleistocene human history of the Tibetan Plateau based on archaeological studies *3*
Section 2: Baishiya Karst Cave *5*
Section 3: Zoological reference samples for an extended ZooMS database *10*
Section 4: Taxonomic assignments *13*
4.1. Morphological taxonomic analysis 13
4.2. ZooMS taxonomic analysis 16
4.3. Comparing and combining morphological and ZooMS results 18
4.4. Palaeoproteomics of the Xiahe 2 specimen 26
Section 5: Skeletal element profiles, faunal taphonomy, and hominin behaviours *29*
5.1. Skeletal element profiles 29
5.2. Faunal taphonomy 31
5.3. Processing of animal carcasses by hominins 39
Section 6: Deamidation *44*
Section 7: Possible subdivisions of layer 10 *50*
References *52*

Other supplementary materials for this manuscript include the following:
Supplementary Dataset 1.
Late Middle and Late Pleistocene sites in East Asia.

Supplementary Dataset 2.
Radiocarbon and deamidation results of bones from T2.

Supplementary Dataset 3.
Collected reference samples for MALDI-ToF MS and LC-MS/MS analyses.

Supplementary Dataset 4.
Database of ZooMS peptide marker masses and peptide marker sequences.

Supplementary Dataset 5.
Taxonomic assignments per layer. A. Morphological taxonomic identifications. B. ZooMS taxonomic identifications. C. Taxonomic identifications after combining morphological and ZooMS taxonomic identifications.

Supplementary Dataset 6.
File in .fasta format containing consensus sequences derived for the Xiahe 2 specimen after shotgun proteomic analysis, and used for phylogenetic analysis.

Section 1: Middle and Late Pleistocene human history of the Tibetan Plateau based on archaeological studies

The Tibetan Plateau (hereafter TP) is the largest high-altitude plateau in the world. As one of the most challenging terrestrial habitats on the earth, it is a crucial area for understanding how biological communities, including humans, adapted to extreme environments. Current archaeological studies reveal a considerably long human occupation history of the TP, hinting at the great potential of studying the adaptation strategy of prehistoric humans to high-altitude environments, both biologically and behaviorally. Recent studies show that Denisovans were present on the TP at least 160 thousand years ago (ka) (Xiahe mandible, named Xiahe 1 in this paper) and continuously or intermittently occupied Baishiya Karst Cave (3,280 meters above sea level [m.a.s.l.], hereafter BKC) at ~100 ka, ~60 ka, and possibly up to ~45 ka[3,4]. Further evidence for hominin presence on the TP during the Middle Pleistocene possibly derives from hand and foot prints at Quesang (4,270 m.a.s.l.), dated to ~226-169 ka[73], and the Piluo site (3,750 m.a.s.l.) on the eastern plateau, dated to a minimum of 130 ka[74]. Although not fully reported, the stone tool assemblage from the Piluo site includes Acheulean-like handaxes and simple core-and-flake products[74]. In contrast, BKC stone artefacts are reported as representing simple core-and-flake technology[4]. Similar stone tool technology is present at the Jiangjunfu 01 site (2,763 m.a.s.l., hereafter JJF01), which is also located on the northeastern TP and dated to ~120-90 ka[7]. The earliest evidence for anatomically modern humans (*Homo sapiens*) on the TP is believed to be represented by the Nwya Devu site (~4,600 m.a.s.l.) in the central TP, dated to ~40-30 ka, which has yielded abundant blade tool technology products[75]. However, no hominin fossils are so far reported from Nwya Devu. Other archaeological sites on the TP with reliable dating results within the Late Pleistocene are largely restricted to the Last Deglaciation period (~18-11.6 ka). These sites contain microblade technology products, which

became increasingly frequent in northern China since about 27 ka[76] and began to appear on the northeastern TP at about 15 ka[77]. Examples include the 151 site (~15-13 ka)[7], Jiangxigou 1 (~15-14 ka)[78] and Heimahe 1 (~13 ka)[78] in the Qinghai Lake Basin (~3,200 m.a.s.l.). Given the relatively young age and the well-developed microblade technology, these assemblages and sites are widely believed to be securely associated with *H. sapiens*.

As an unusual Palaeolithic site on the TP with a large number of faunal remains and a long-term human occupation history, BKC provides a unique opportunity to study Denisovan behaviours on the TP through the analysis of well-preserved faunal remains. Previous publications about BKC mentioned the presence of morphologically identifiable specimens of woolly rhinoceros (*Coelodonta* sp.), gazelles (*Procapra* sp.), foxes (*Vulpes* sp.), spotted hyena (*Crocuta* sp.) and marmots (*Marmota* sp.), including the presence of cut and percussion marks on several of these species[4]. In contrast, JJF01 has so far produced a small bone assemblage of 11 specimens[7]. These include one herbivore tooth fragment and some specimens likely deriving from middle to large-sized mammals. The fragmentary bone specimens found at sites dated to the Last Deglaciation could not be efficiently identified by zooarchaeological analysis[78]. Only the lower cultural layer of the 151 site allows for the identification of 126 faunal specimens. These consist of large ungulates (90%), including *Bos* sp. and *Equus* sp., small ungulates (Przewalskii's gazelle-size) (9.5%), and one individual of woolly hare (*Lepus oiostolus*)[8]. Besides these two reports, no further evidence is available on the late Middle to Late Pleistocene faunal community composition on the northeastern TP. Therefore, we currently know very little about faunal community and hominin subsistence strategies on the TP during the occupations of archaic hominins and early modern humans.

Section 2: Baishiya Karst Cave

Baishiya Karst Cave (35.45° N, 102.57° E) is located in the Ganjia Basin, northeastern TP. It is a karstic cave and lies ~20 m above the riverbed of Jianglagou river. In 1980, an anonymous monk recovered a hominin hemimandible (Xiahe 1) in this cave. Unfortunately, we do not know exactly where the mandible is from within this complex and big cave. U-series dating of the adhering carbonate crust on the outside of the mandible provide a minimum age of about 160 ka for the mandible[3]. Ancient hominin DNA was not recovered from this specimen, while palaeoproteomic analysis indicates it belongs to Denisovans[3]. The Xiahe 1 mandible provides abundant morphological characteristics of Denisovans. Based on some similar anthropological traits, Denisovans at BKC might have closer relationships with some known archaic hominin fossils in East and Southeast Asia, for example, Penghu 1 and Xujiayao specimens[3], the Harbin specimen[79], and the reported Denisovan-like molar from northern Laos[2].

In 2018 and 2019, our team excavated Baishiya Karst Cave and collected numerous lithics and bone fossils in two connected 1 m × 2 m excavation units (T2 and T3). The stratigraphy of these two units is consistent. The stratigraphic descriptions and dating of layer 2 to the middle of layer 10, excavated in 2018, were reported previously[4]. Additionally, the analysis of Denisovan mitochondrial DNA (mtDNA) from sediments from layers 7 and 4 indicate Denisovans occupied the cave at ~100 ka, ~60 ka, and possibly as late as 45 ka[4]. In addition, Denisovan mtDNA was also recovered from layers 2 and 3[4]. This provides additional evidence of Denisovan occupation at BKC, besides the Xiahe 1 mandible.

In 2019, the complete layer 10 and the new layer 11 were uncovered both in T2 and T3 (SI Fig. 2.1). During excavation, layer 10 was further subdivided into 10a (~20-50 cm thick), 10b (~10-40 cm thick), 10c (~5-30 cm thick), 10d (10-30 cm thick), based on the variation of sedimentation traits. Layer 11, ~10-20 cm thick, is mainly composed of pebbles, lithics,

fragmented bones and calcite crystals. Archaeological excavations are ongoing at the site and more layers below layer 11 will be uncovered in the future.

Of note are two historical bowl-shaped pits (H1 in T2 and H3 in T3)[4]. Both pits break layer 2 to middle layer 10, taking ~30% to 70% of the whole excavated area in these layers. As a result, the number of bone specimens recovered from the archaeological layers varies greatly in accordance with the breakage extent of the historical pits (SI Table 2.1). Besides a few pieces of historical period pottery sherds and faunal remains, H1 and H3 also contain a considerable number of prehistoric stone artefacts and faunal remains.

The chronological framework for layers 2-10 of T2 has been established based on optically stimulated luminescence (OSL) and radiocarbon dating results[4] (Supplementary Dataset 2). Here, we apply this chronology to both T2 and T3, and choose the maximum ranges of the modelled ages to represent the age of each layer (SI Table 2.2). Radiometric dating of layer 11 is in progress. Available evidence suggests that layer 10 formed at the latest by 109 ka. Layers 9 to 6 accumulated in quick succession between approximately 120 ka and 90 ka. Considering the lack of direct dating for layer 5, we used the age interval between layers 4 and 6 as a substitute for the true age of layer 5. In addition, we must note that the previous chronological analysis has raised the possibility that layer 4 can be subdivided into an older component, dating to ~66 ka, and a younger component, dating to ~47 ka (SI Table 2.2). However, these two components cannot be separated stratigraphically in the field. Bone materials from this layer can therefore correspond to different chronological episodes. Layer 3 is dated to between 48 and 32 ka, while layer 2 seems to last from 32 ka to 5 ka[4].

We collected a total of 3,642 bone specimens from T2 and T3 during excavations in 2018 and 2019. Largely based on a minimum length of 20 mm, we selected 2,567 specimens for zooarchaeological analysis, of which 1,857 specimens were sampled for ZooMS

(Zooarchaeology by Mass Spectrometry) analysis. Details about the samples can be found in the "Sample selection" section in the Methods and in SI Table 2.1.

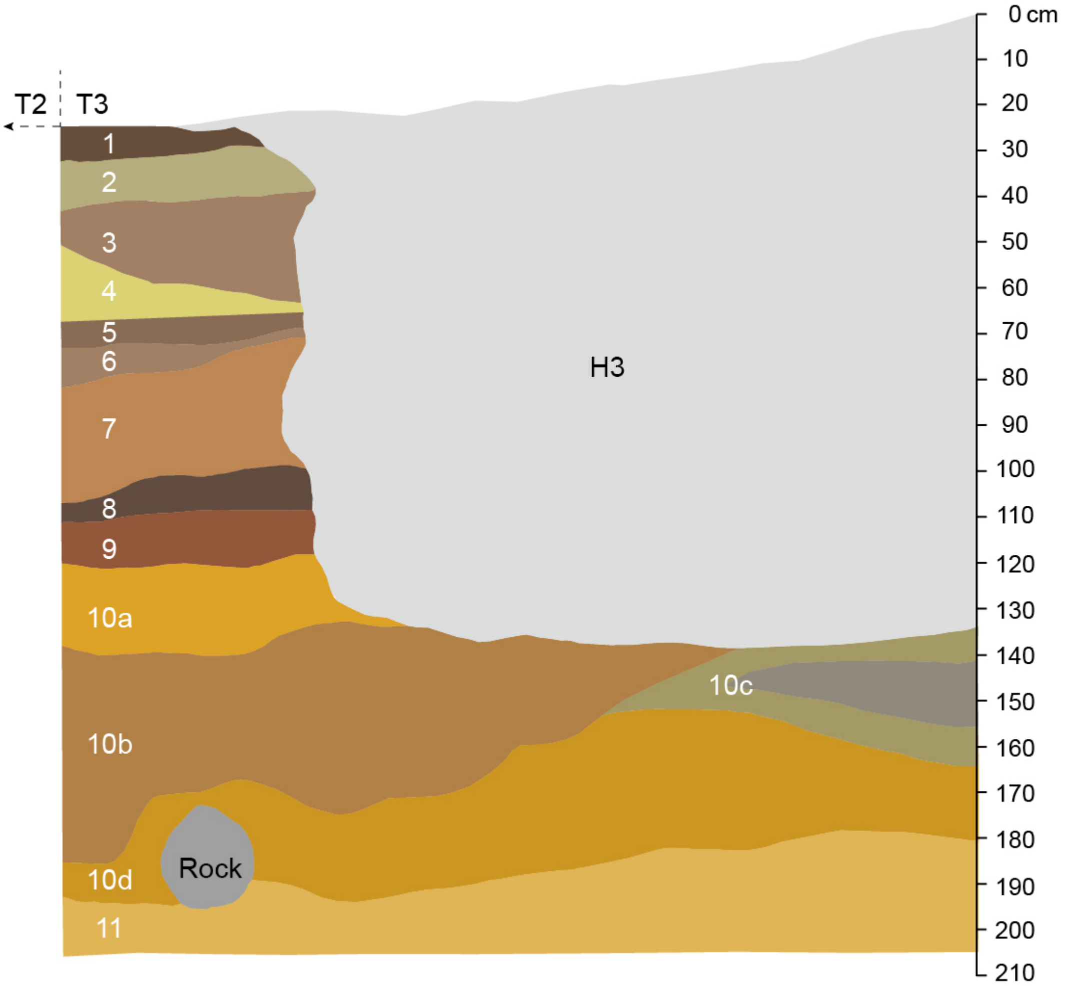

SI Fig. 2.1. Section drawing of the southwest wall profile in T3. Layers 1 to 11, and the historical pit feature (H3) are indicated.

SI Table 2.1. Bone samples from BKC selected for morphology and ZooMS analyses in this study. The column "Morphology" refers to the total number of specimens analysed through zooarchaeological methods, including morphological taxonomic analysis, weathering stages, bone surface modifications, and so on. The column "ZooMS" refers to the number of specimens selected from the morphologically analysed specimens for further proteomic analysis. The final column, "Morphologically identified specimens for ZooMS" indicates the number of specimens with morphological taxonomic identifications that were selected for proteomic analysis, to confirm their morphological taxonomic assignments.

Layer	Morphology	ZooMS	Morphologically identified specimens for ZooMS
**1**	42	11	0
**2**	55	19	3
**3**	220	156	6
**4**	115	82	4
**5**	97	59	2
**6**	63	49	1
**7**	83	65	3
**8**	22	15	0
**9**	67	59	2
**10**	713	494	23
**11**	930	757	5
**Pits (H1 and H3)**	138	73	4
**Unknown**	22	18	0
**Total**	**2,567**	**1,857**	**53**

SI Table 2.2. The stratigraphic chronology applied in this paper.

Chronological pattern of T2 (Table S12)[4]					In this paper				
Layer	From (95.4%; ka)	To (95.4%; ka)	Mean (ka)	±	Layer	Min (ka)	Max (ka)	Mean (ka)	±
**End Layer 2**	5.4	28.4	16.9	11.5					
					**2**	5.4	34.2	19.8	14.4
**Transition 3/2**	32.1	34.2	33.2	1.1					
					**3**	32.1	48.1	40.1	8.0
**Start Layer 3**	43.8	48.1	46.0	2.2					
**End Layer 4**	44.7	49.2	47.0	2.3					
					**4**	44.7	72.0	58.4	13.7
**Start Layer 4**	59.9	72	66.0	6.1					
					**5**	59.9	102.0	81.0	21.1
**End Layer 6**	90.6	102	96.3	5.7					
					**6**	90.6	104.1	97.4	6.8
**Start Layer 6**	95.2	104.1	99.7	4.5					
**End Layer 7**	96.6	105.8	101.2	4.6					
					**7**	96.6	107.7	102.2	5.6
**Start Layer 7**	97.9	107.7	102.8	4.9					
**End Layer 8**	99	109.9	104.5	5.5					
					**8**	99.0	113.2	106.1	7.1
**Start Layer 8**	100.7	113.2	107.0	6.3					
**End Layer 9**	103.4	116.9	110.2	6.8					
					**9**	103.4	149.2	126.3	22.9
**Transition 10/9**	109	149.2	129.1	20.1					
					**10**	109.0	>224.8	>166.9	57.9
**Start Layer 10**	156.9	224.8	190.9	34.0					
					**11**	In progress	In progress		

Section 3: Zoological reference samples for an extended ZooMS database

We successfully obtained reference MALDI-ToF MS spectra for 39 species and collagen type I sequences for 14 species (Supplementary Dataset 3). Based on these new data and previous datasets[19,80-84], we can confirm predicted peptide markers for species like Tibetan antelope (*Pantholops hodgsonii*) and Przewalski's gazelle *(Procapra przewalskii*). Additionally, our dataset provides COL1 sequences and peptide marker mass series for species in and around the TP, such as the takin (*Budorcas taxicolor*) and the mainland serow (*Capricornis sumatraensis*).

In addition, we derived an extended peptide marker database of 5 additional peptide markers to effectively distinguish mammals to subfamily or genus levels (Supplementary Dataset 4). As a result, our analysis and ZooMS reference database now includes 362 species entries for a total of 24 peptide markers. The five newly-added peptide markers are informative in the following taxonomic contexts (m/z values are given as rounded numbers, see Supplementary Dataset 4 for accession numbers, common names and Latin names, peptide amino acid sequences and m/z values):

1. COL1α1 -8-9. The COL1α1 -8-9 separates some early-diverging cats (including all Pantherinae) and the ocelot lineage (with a peak at m/z 1,523+1,539) from late-diverging small cats (genera *Felis*, *Lynx*, *Puma*, *Acinonyx*, *Prionailurus*, with a peak at m/z 1,553+1,569).
2. COL1α1 271-291. For most mammals in the database, the peak of COL1α1 271-291 is at m/z 2,020. For Leporidae and Moschidae, the peak is at m/z 2,006, and therefore distinguishes Moschidae from other Pecora.
3. COL1α1 733-756. This peptide is with one missed cleavage in the position COL1α1 740, with m/z of peaks at 2,216 and 2,232 for most Bovidae. While the peak of this

peptide in *Procapra* sp. is at m/z 2,188 and 2,204, which is useful for separating *Procapra* from other Antilopinae. The substitution of Proline (P) with Alanine (A) introduces a new cleavage site for some genera (*Ovis*, *Budorcas*, *Rupicapra*, *Capricornis*, *Naemorhedus*), generating low-mass m/z values (744 and 1,448, or 2,174+2,190) in the spectra of these genera. While other genera of Caprinae (*Capra*, *Pantholops*, *Pseudois*) have m/z values of 2,216+2,232 for the same peptide. The above information helps to further distinguish Caprinae at the genus level. This peptide also efficiently distinguishes between *Crocuta* sp. (m/z 2,246 +2,262) and Pantherinae (m/z 2,216 +2,232).

4. COL1α2 859-884. The COL1α2 859-884 has multiple peaks in Bovidae and distinguishes Cervidae and *Gazella* sp., with the peaks at 2,293 and 2,261 respectively.
5. COL1α1 889-906. The peak of COL1A1 889-906 for Equidae is at m/z 1,530, which is different from other mammals in the database. It is useful to distinguish the Equidae in cases where higher-molecular masses are absent or of low intensity.

Notably, some peptide markers such as COL1α1 -8-9 and COL1α2 859-884, as well as COL1α2 375-396 reported by Janzen et al.[82], are not observed in the Pleistocene bone remains of the BKC, but are present in samples with deamidation values close to 1 from the historical pits and several of the reference specimens used during database expansion. These peptides might therefore be diagenetically altered and/or lost in many Pleistocene bone proteomes. Finally, by adding new data of species from TP, we provide a new ZooMS marker database covering 362 mammalian species, including confirmed peptide markers and genetically predicted peptide markers (Supplementary Dataset 4). This resource will be of use for future ZooMS and proteomic[85] studies on the TP and other palaeontological and archaeological sites in the world.

SI Table 3.1. Specified gradient for Easy NanoLC from Thermo Fisher Scientific (MA).

Time (min)	Duration (min)	%B
0	0	5
50	50	30
60	10	45
62	2	80
67	5	80
72	5	5
77	5	5

Section 4: Taxonomic assignments

Traditionally, zooarchaeology can identify some skeletal remains with taxon-specific morphological characteristics to genus or species levels, but generally this is only possible for 20-30% of the remains, or less, in Pleistocene faunal assemblages. At the same time, zooarchaeology also reveals taphonomic processes and hominin behaviours by observing and recording bone surface modifications. As taxonomic information is lacking for the vast majority of bone remains excavated, only a small fraction of anthropogenic surface modifications is therefore directly associated with taxonomic information, limiting some of the ecological insights into hominin behaviours.

In contrast to morphological observations, ZooMS is an effective method to identify fragmented bone specimens that cannot be morphologically identified[80]. It is a relatively low-cost, quick and precise method to identify bone fragments up to subfamily or genus level[86] and has been used to screen hominin bones from hundreds or thousands of otherwise unidentifiable bone fragments[87-89]. In addition, glutamine deamidation values can be calculated from the same mass spectra used for taxonomic identifications, and potentially provide information about the burial environment of these bone fragments[18,69]. Recent studies have revealed that the integration of ZooMS taxonomic identifications into more traditional zooarchaeological frameworks has the potential to provide enhanced ecological, hominin behaviour, and taphonomic insights[12-16,90-92].

4.1. Morphological taxonomic analysis

The faunal remains at BKC include a large number of bone fragments that are morphologically unidentifiable. Morphologically identifiable specimens are largely derived from crania, individual teeth, epiphyses, and cubic bones. In total, 661 bone specimens (25.75% of 2,567) from at least 149 individuals are identified by morphology, to 30 different taxonomic groupings

(Supplementary Dataset 5A). This faunal assemblage includes globallly extinct and locally extinct animals (e.g. woolly rhinoceros [*Coelodonta* sp.], spotted hyena [*Crocuta crocuta ultima*], and porcupine [*Hystrix* cf. *subcristata*]), species endemic to the TP (e.g. wild yak [*Bos* cf. *mutus*], snow leopard [*Panthera* cf. *uncia*], Tibetan fox [*Vulpes ferrilata*], Himalayan marmot [*Marmota himalayana*] and *L. oiostolus*), and species common in the Palaearctic realm today (e.g. equids [*Equus* sp.], red deer [*Cervus elaphus*], and wolf [*Canis lupus*]) (Supplementary Dataset 5A). The assemblage represents a diverse range of Middle and Late Pleistocene mammals on the northeastern TP, and provides continuous data on mammal community composition during this period.

The vast majority of the fossils recovered in BKC belong to several bovid species, mainly Caprinae followed by *Bos* sp. (Supplementary Dataset 5A). In general, Caprinae (~32%, calculated from NISP results, same hereafter), including bharal (*Pseudois nayaur*) and argali (*Ovis ammon*), are the most abundant taxon at the BKC, and present in all stratigraphic units except layers 8 and 9, where only 6 and 11 specimens, respectively, could be identified in total. Other ungulates, including *B.* cf. *mutus* (8.61%), Tibetan gazelle [*Procapra* cf. *picticaudata*] (10.73%), *Equus* sp. (6.80%), musk deer [*Moschus* sp.] (1.66%) and *Cervus* sp. (3.32%), as well as carnivores, including *C. lupus* (1.57%), *V. ferrilata* (2.87%), *C. crocuta ultima* (5.14%), *P.* cf. *uncia* (0.15%), and beech marten [*Martes* cf. *foina*] (1.36%), are also present in some layers. In addition, small animals (19.34%), like rodents (*H.* cf. *subcristata*, Gansu zokor [*Myospalax cansus*], groove-toothed flying squirrel [*Aeretes melanopterus*] and voles [*Microtus* sp.]), lagomorphs (*L. oiostolus* and pikas [*Ochotona* sp.]), and birds (golden eagle [*Aquila chrysaetos*], common pheasant [*Phasianus colchicus*], quails [*Coturnix* sp.] and owls [Strigiformes]), are present throughout all layers. Moreover, we note that extinct megaherbivores and large carnivores, such as *Coelodonta* sp. and *C. crocuta ultima* only appear in older stratigraphic units (layers 10 and 11). The locally extinct porcupine, *H.* cf. *subcristata*

(*n* = 1), also appeared in layer 10a. Overall, the remains of vertebrate fauna suggest that a high diversity of biotopes persisted in the vicinity of the cave.

The faunal assemblage from the BKC can reflect the surrounding environment to a certain extent. It contains some mammals typical of the steppe biotopes, such as *Coelodonta* sp., *Equus* sp., *B.* cf. *mutus*, *P.* cf. *picticaudata* and the extinct predator *C. crocuta ultima*. It also contains some typical representatives of mountain-steppe biotopes, such as *O. ammon* and *P. nayaur*, as well as other representatives of open landscape biotopes, such as, *L. oiostolus* and *M. himalayana*. In addition, some fauna typical of forest-shrub environments are also present in this assemblage, including *C. elaphus*, *Moschus* sp., *H.* cf. *subcristata* and *A. melanopterus*. Overall, a significant number and variety of fauna associated with open landscapes (steppe biotopes: 3.6-38.6% and mountain-steppe biotopes: 24.4-69.1%) from layers 11-2 indicate a generally stable, open environment in the Ganjia Basin during the Middle and Late Pleistocene. Meanwhile, the presence of some species characteristic of a forest-shrub biotope (6.3-18.0%), suggest the existence of small-scale mosaic forest-shrub landscapes.

The faunal assemblage from BKC also provides additional data for exploring Denisovan habitats. The mix of an open grass-dominated landscape with patches of forest-shrubs indicated by the BKC faunal assemblage is similar to the faunal information available for Denisova Cave, Russia. However, there high-latitude open tundra also exists, as evidenced by the presence of reindeer (*Rangifer tarandus*) in its faunal assemblages[93]. In addition, another most likely Denisovan site, Tam Ngu Hao 2 (Cobra Cave, Laos), has recently been described in the (sub)tropical regions of southeast Asia. The associated fauna from Tam Ngu Hao 2 suggests the presence of open forests and savannah landscapes surrounding the locality[94]. Together with the new evidence presented from BKC, this indicates that Denisovans were capable of adapting to a highly diverse range of mosaic open environments, from temperate

habitats in the Altai, the high-altitude conditions on the TP, and even to the (sub)tropical habitats of southeast Asia.

4.2. ZooMS taxonomic analysis

According to the updated database (Supplementary Dataset 4), ZooMS analysis resulted in 74.42% (1,382/1,857) successful identifications, comprising a total of 23 taxonomic groups (Supplementary Dataset 5B). At BKC, almost all sampled bone specimens within layers 1-7 could be identified by ZooMS, while the percentage of identifiable specimens within layers 10 and 11 is approximately 60-70%, which is due to poor collagen preservation in these older layers. For the same reason, about 10% of the spectra of layers 10-11 can be assigned to order or sub-order levels only, such as Bovidae/Cervinae and Bovidae/Cervinae/Moschidae. Of note is that most of the specimens (~90%) from layers 8-9 failed for ZooMS analysis. This is probably either due to some post-depositional taphonomic process or high-temperature heating of the bones prior to deposition in the archaeological sediments.

Our ZooMS identifications are based on the 24 peptide markers present in the updated database (Supplementary Dataset 4). Some taxonomic groups are difficult to be separated due to the absence of discriminating peptide markers, such as *Gazella* sp. and Cervinae, whose peptide markers are highly similar. Although we identified the novel peptide marker COL1α2 859-884, which could efficiently distinguish them. Unfortunately, this peptide is not present in the Pleistocene spectra of BKC. Similarly, we cannot distinguish between *Bison* sp. and *Bos* sp. by ZooMS. However, of the six *Bos* sp./*Bison* sp. specimens screened from BKC by ZooMS, four specimens were further identified as possibly *B. mutus* by SPIN (Species by Proteome INvestigation) analysis[95], which is consistent with the morphological identification results. Moreover, considering the distribution range of *Bison* sp. in Eurasia[96], we can exclude it from the faunal composition at BKC.

The "Caprinae" category (n = 466) comprises *Pseudois*/*Hemitragus* (n = 279), *Pseudois*/*Hemitragus*/*Capra* (n = 118), *Pseudois*/*Hemitragus*/*Ovis*/*Capra* (n = 33) and *Pseudois*/*Hemitragus*/*Ovis* (n = 18). For *Hemitragus* sp., which is nowadays only naturally distributed in western TP, we only have the mass information for the peptide marker COL1a2 757-789 (G/G')[97]. Due to this, we cannot exclude this species from the ZooMS categories. Although some low-quality spectra lacking the peptide marker COL1a2 757-789 were identified to the category including *Capra* sp., we did not find any peaks with m/z at 3,077+3,093, which is a unique marker of *Capra* sp. compared to other species of Caprinae. Therefore, considering additional information, like geographical distribution and morphological results, the classification of Caprinae identified by ZooMS is mainly *Pseudois* sp. The genus *Pseudois* comprises two living species, the bharal (*P. nayaur*) and the dwarf bharal (*P. schaeferi*)[98], although there is growing evidence that the two species can be considered conspecifics[99]. As the most typical species of genus *Pseudois, P. nayaur* occupies a variety of habitats across the TP, primarily between 2,500 to 5,500 m.a.s.l., including forest, shrub, grassland, and deserts, while its main presence is in rocky areas (e.g. inland cliffs, mountain peaks)[100]. Therefore, they are highly tolerant of environmental extremes, from hot desert mountains to cold mountain slopes[101].

In addition, according to the peptide markers described in Buckley et al.[80] and Eda et al.[102], birds (Avian) were identified by ZooMS. They are present in most layers. However, due to the lack of an in-depth reference database for avifauna we did not classify them to a higher taxonomic level.

The ZooMS identifiable bone assemblage is dominated by remains of Caprinae (33.72%), *Bos* sp. (16.57%), Equidae (12.45%), and Cervinae/*Gazella* sp. (12.30%) (Supplementary Dataset 5B). In addition, we identify remains of various carnivores (Felinae, *Crocuta* sp., Felinae/Ursinae, Pantherinae, Carnivora, Ursinae, Canidae but not *V.*

vulpes/*ferrilata*, *V. vulpes*/*ferrilata*; 2.17%), megaherbivores (Rhinocerotidae; 3.04%), and small mammals (*Marmota* sp., Leporidae and birds; 2.17%). In particular, unlike morphological results, the Rhinocerotidae and large carnivores remain present up to layer 6. The implication is that these megafauna were present in the Ganjia Basin at least until the beginning of the Last Glacial Period at BKC.

4.3. Comparing and combining morphological and ZooMS results

By comparing the taxonomic groups identified by the two taxonomic identification methods, we found that the ZooMS taxonomic groups are also represented among the morphological results (Supplementary Dataset 5). Exceptions to this are Ursidae, Felinae, and *Pantholops* sp., which had extremely low proportions (*n* = 0.07%; *n* = 0.07%, and *n* = 0.22%, respectively) in the ZooMS dataset. Considering that highly fragmented bones limit the opportunity of precise morphological identifications, some species present at low frequencies may be missing in the faunal composition of the morphologically identified dataset. This would also explain the absence of megafauna in layers 6 and 7 in the morphological results but their presence in these two layers in the ZooMS results. Meanwhile, some small animal species identified by morphology, such as Mustelidae, and Rodentia (except *Marmota* sp.) and *Ochotona* sp., are absent in the ZooMS results. This is probably mainly the result of sample selection. As previously mentioned, ZooMS identification generally focused on fragmented bone specimens longer than 20 mm, so small animal specimens were disfavoured during sample selection for ZooMS analysis.

At BKC, we found that most of the specimens with lengths greater than 100 mm could be identified by morphological analysis (SI Fig. 4.1a). This is due to the fact that the larger specimens (>100 mm) retain more morphological traits. In contrast, specimens smaller than 20 mm are identified mainly by morphological analysis. This is due to the deliberate selection of

specimens with morphological characters within specimens of this length range. In addition, only a small number of specimens smaller than 20 mm were selected for ZooMS analysis, resulting in the small number of identified specimens smaller than 20 mm (SI Fig. 4.1a).

As shown in Fig. 1b, it is evident that morphologically identifiable specimens are largely derived from head fragments, carpals, tarsals, and associated foot bones. The application of ZooMS has increased our taxonomic identifications, allowing us to successfully identify a larger number of axial and front/hind limb shaft fragments. It is well-known that identifying axial elements is generally problematic in highly fragmented archaeological faunal assemblages. They are usually underrepresented in skeletal profile representations, leading to potential misinterpretations regarding past hominin carcass transport strategies. Therefore, the combination of ZooMS and traditional morphological taxonomic identification approaches provides a potentially more complete view of hominin subsistence behaviour related to carcass transport.

Bone surface modification traces are distributed mostly on limb bone shaft fragments and axial bone fragments (Extended Data Fig. 4a), which are difficult to identify morphologically (Fig. 1b). However, ZooMS can effectively make up for this shortage, and increases the number of identified specimens with hominin modifications significantly, from 81 to 386 (Extended Data Fig. 4b). This has improved our understanding and knowledge of the carcass butchering activities of different taxa. It also enables a more detailed study of anthropogenic aggregation of faunal assemblages and processing of carcass resources by hominins, a level of detail not achievable for BKC through traditional morphological analyses alone. For example, most of the front/hind limb diaphyses with anthropogenic modification traces can only be identified to body-size in a traditional zooarchaeological approach, whereas ZooMS analyses allow for further taxonomic assignments of such bone specimens.

In addition, we find that the body-size classes recorded in the morphological dataset are hard to match with the taxonomic identities established using ZooMS (SI Fig. 4.1b). For example, the body-size class of *Bos* sp. should belong to Class IV, but the taxon appeared in all body-size classes from size class II to size class IV/V. Therefore, as previous studies have shown[12,14], it is biased to allocate unidentified bone fragments into a certain taxonomic group according to the body-size class observed by morphology.

By comparison, we note that combining morphological and ZooMS taxonomic identifications enriched the composition of the faunal assemblage in each stratigraphic unit, although general proportional trends remain (Extended Data Fig. 3). With additional ZooMS results, the number of identified specimens at BKC improved from 661 to 2,005 (Supplementary Dataset 5C). In addition, due to morphological traits being rather similar among medium-sized bovids, ZooMS helps to confirm specific species, such as the assignment of most *Ovis*/*Pseudois* specimens to the genus *Pseudois*. Meanwhile, the morphological results also help to specify ZooMS taxonomic groups with more precision. For example by demonstrating that all specimens identified as "Canidae (not *V. vulpes*/*ferrilata*)" likely belong to *C. lupus*. All of these improved our understanding of the faunal assemblage, and hominin behaviours, at BKC.

After integrating ZooMS identification results into the morphologically identified dataset, a total of 2,005 specimens have been identified to specific taxonomic groups (Supplementary Dataset 5C). The proportion of different taxa is similar to the previous ZooMS or morphological results, all reflecting that medium-sized mammals, like Caprinae (33.02%), dominated the faunal assemblage. Except for the Tibetan antelope (*Pantholops* sp.), which only appears in layers 10-11, skeletal specimens of other artiodactyla include Caprinae (33.02%), *Bos* sp. (15.06%), Cervinae/*Gazella* sp. (9.58%), *Procapra* sp. (4.14%), Moschidae (1.35%) and perissodactyla (Equidae, 10.67% and Rhinocerotidae, 2.99%) were consistently present in

most layers at BKC. Small-sized animals, like *Marmota* sp. (1.70%) and Leporidae (3.14%), and birds (2.24%) also appear in almost all layers. Carnivores accounted for 5.09% of the combined (ZooMS + morphological identifications) dataset, which is a significant increase compared to the ZooMS results (2.17%). This is due to the fact that the remains of the *Crocuta* sp. in layer 10 are more complete than those of associated herbivores, thus greatly increasing the NISP of carnivores (NISP increases from 30 to 97) after the inclusion of the morphological identification results.

In addition, megaherbivores (Rhinocerotidae, 2.99%) are only identified in layers 6, 7 and 10, though overall sample sizes of younger layers are not as large as of layer 10 (Supplementary Dataset 5C). The disappearance of woolly rhinoceros might suggest a significant change in faunal community composition in the Ganjia Basin during the period when layer 6 and layer 5 formed (end of MIS 5), as megaherbivores possess key functional traits that can affect a variety of species within an ecosystem[103]. This shift coincides with a decrease in temperature (Fig. 4a) and monsoon rainfall intensity (Fig. 4b and 4c), which suggests that climatic changes might have played a role in the local extinction of the woolly rhinoceros. However, this shift may also be related to changes in hominin foraging strategies. According to the fossil records[104] and sedimentary DNA studies[105], the extinction age of woolly rhinoceros in Eurasia is around 10 ka, much later than its last documented appearance in the Ganjia Basin. In addition, large carnivores (e.g. *Crocuta* sp., Ursinae, Pantherinae), also only appear in layers 7, 10 and 11 in BKC, similar to megaherbivores (Supplementary Dataset 5C). However, some of them, such as *P. uncia*, though they are not found in younger layers (layers 1-6) at BKC, are still present in the northeastern TP today[106]. Hence, without further evidence, it is hard to discuss the exact disappearance time and reason of these species in Ganjia Basin in this paper.

It is noteworthy that only 8 and 18 faunal specimens are identified from layers 8 and 9, respectively, limiting our understanding of the faunal community composition during the period when these layers formed. Meanwhile, the bone remains from layer 1 and the two historical pits (H1 and H3) were highly influenced by recent human activities. Therefore, to better reconstruct faunal community composition during the late Middle and Late Pleistocene at BKC, we created a new dataset (Extended Data Table 1), which only includes clearly classified mammal taxonomic groups from layers 2-7 and 10-11. According to this new dataset, it is evident that the proportion of Caprinae increased over time, while *Bos* sp. has an opposite trend (Extended Data Fig. 3 and SI Fig. 4.2). The trend of other common groups at BKC, such as *Procapra* sp. and *Cervus* sp., shows no significant difference across layers.

In addition, Simpson and Shannon indices were also calculated based on Extended Data Table 1. For both indices, higher values suggest higher taxon diversity. Although Simpson and Shannon indices emphasise different components of diversity (evenness and richness, respectively), they have a similar trend across the stratigraphy of BKC, both reflecting a slight decrease in taxonomic biodiversity from layers 10 to 2, but with little variation between layers (SI Fig. 4.3).

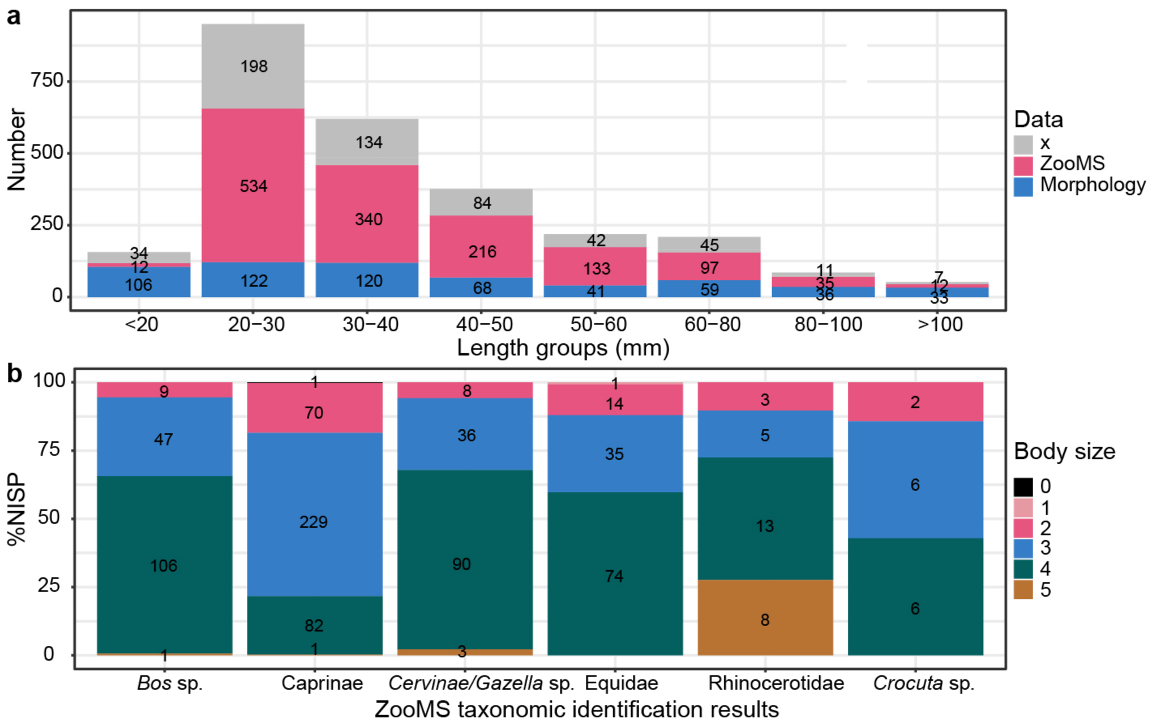

SI Fig. 4.1. The comparison and combination of the taxonomic identification results between morphology and ZooMS. (**a**) Taxonomically identified specimens and their bone length distributions. "x" represents specimen counts not taxonomically identified through either morphological observation or ZooMS analysis. (**b**) Comparison between ZooMS taxonomic identification results (Supplementary Dataset 5B) and the body-size classes recorded by morphology. For **a** and **b**, the number displayed in each bar segment is the corresponding NISP count.

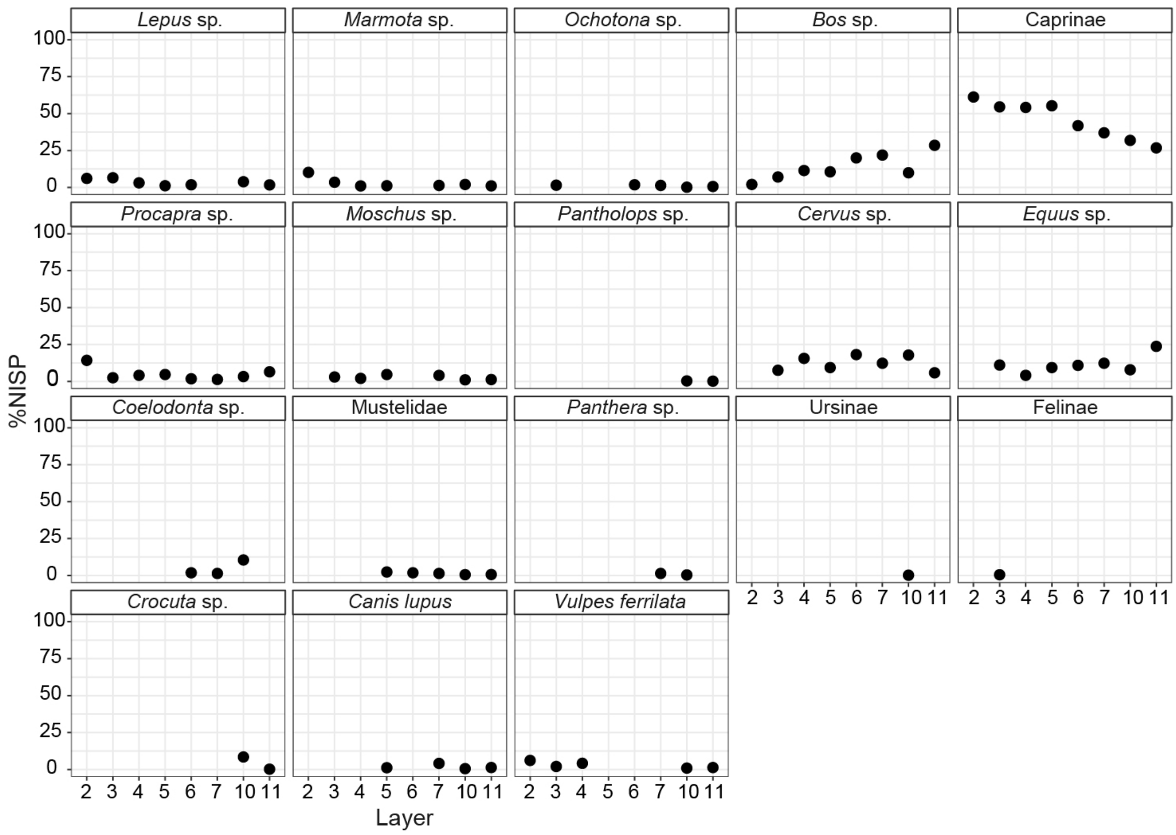

SI Fig. 4.2. The percentage of identifiable specimens of selected taxonomic groups (Extended Data Table 1) in each layer.

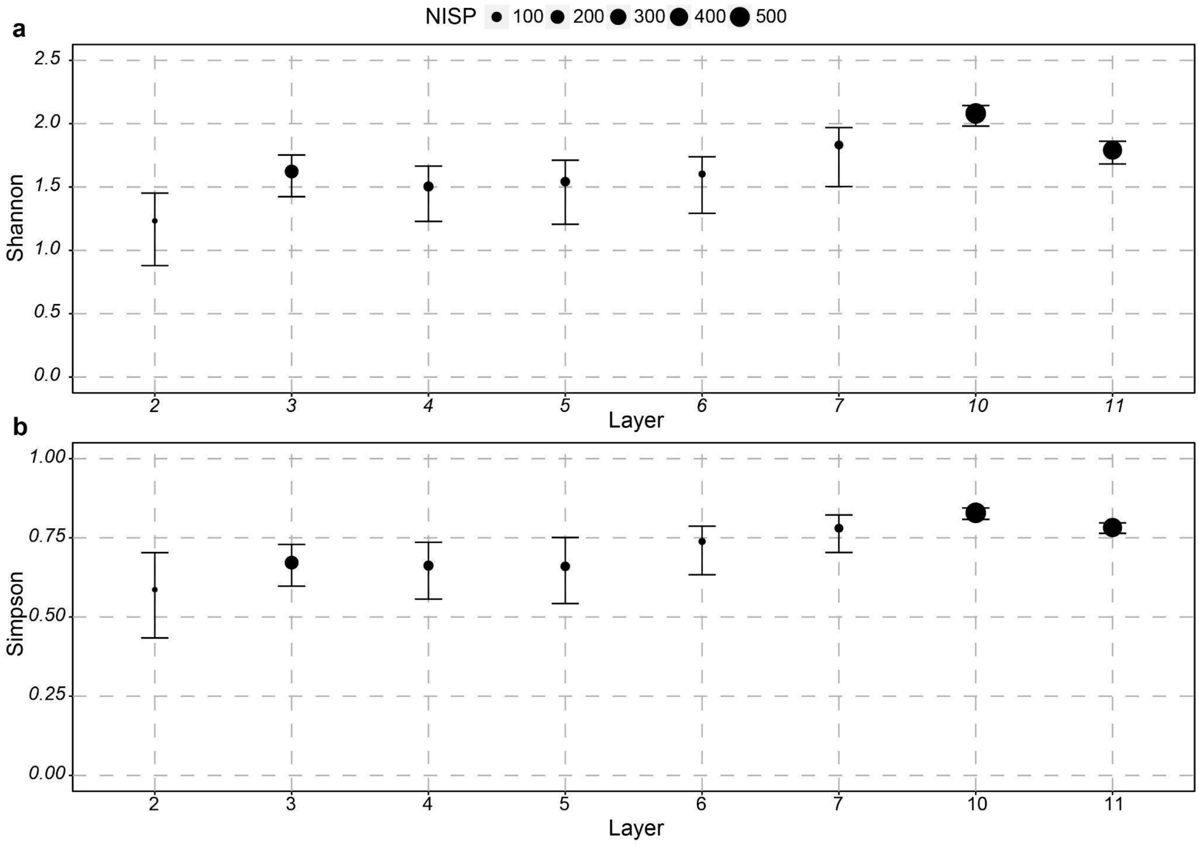

SI Fig. 4.3. Diversity indices calculated for BKC faunal assemblages. (**a**) Shannon index, (**b**) Simpson index. Point size is proportional to the NISP. The bar range represents bootstrap confidence intervals, with the top and bottom lines of the bar ranges representing the 2.5% and the 97.5% bootstrap quantiles, respectively.

4.4. Palaeoproteomics of the Xiahe 2 specimen

A total of 21 non-contaminant proteins had five or more peptides assigned, and were therefore reconstructed for phylogenetic analysis of the Xiahe 2 specimen (SI Table 4.1 and 4.2; Supplementary Dataset 6). Between 33 and 1,056 amino acids were identified per protein, leading to coverages ranging between 2.8% and 74.6% (calculated based on the corresponding modern human protein). All reconstructed proteins were collagens, which is expected, as these proteins make up the majority of the bone proteome, and have been found to preserve well over time in archaeological skeletal elements[107]. The vast majority of the bone proteome consists of the alpha–1 and alpha–2 chains of type I collagen, and these two proteins also have the highest reconstructed coverage in Xiahe 2 (72.1% and 74.6%, respectively).

Compared to the Xiahe 1 specimen[3], the Xiahe 2 reconstructed proteome is much more extensive. The six proteins reconstructed for Xiahe 1 are also reconstructed for Xiahe 2, but at a higher coverage (SI Table 4.1). Despite some differences in extraction, mass spectrometry instrumentation, and protein search strategy, we take this to indicate a better proteome preservation in the Xiahe 2 specimen.

The amino acid polymorphism used to identify Xiahe 1 as a Denisovan, COL1A2 R996K, is also identified in Xiahe 2. Additionally, Xiahe 2 has a Denisovan-specific SAP at COL5A3 P942S, which is not shared with any of the other taxa included in the phylogenetic analysis. However, the unique SAP identified in Xiahe 1, COL2A1 E583G, is not identified in Xiahe 2.

SI Table 4.1. Protein recovery from specimens Xiahe 1[3] and 2. Percent coverage is based on the length of the modern human proteome reference accession protein sequence (see SI Table 4.2).

Protein	Amino acid positions recovered	Xiahe 2 coverage %	Xiahe 1 coverage %[3]
COL1A1	1,056	72.1	61.1
COL1A2	1,019	74.6	54.8
COL2A1	385	25.9	25.2
COL3A1	220	15.0	7.0
COL4A5	65	3.9	-
COL4A6	79	4.7	-
COL5A1	397	21.6	2.6
COL5A2	444	30.0	11.1
COL5A3	86	4.9	-
COL6A2	33	3.2	-
COL7A1	82	2.8	-
COL9A1	87	9.4	-
COL10A1	47	6.9	-
COL11A1	125	6.9	-
COL11A2	132	7.6	-
COL18A1	54	3.1	-
COL21A1	37	3.9	-
COL22A1	45	2.8	-
COL23A1	44	8.1	-
COL24A1	73	4.3	-
COL27A1	87	4.7	-

SI Table 4.2. Protein accessions of all proteins used for phylogenetic analysis of Xiahe 2.

Protein	*Homo sapiens*	*Gorilla gorilla*	*Pongo abelii*	*Pan troglodytes*
COL1A1	P02452	G3RBN8	H2NVM9	H2QDE6
COL1A2	P08123	G3QT97	A0A2J8WJU0	A0A2J8L483
COL2A1	P02458	G3QNV9	H2NH33	H2R193
COL3A1	P02461	G3RK87	H2P837	H2QJ46
COL4A5	P29400	A0A2I2YBW5	H2PWG6	A0A6D2W6K3
COL4A6	Q14031	G3QKF9	H2PWG3	A0A6D2Y535
COL5A1	P20908	G3R760	A0A2J8UIS5	K7CMZ9
COL5A2	P05997	G3RDT1	H2P838	H2R6B8
COL5A3	P25940	G3RH82	H2NXH7	K7BN45
COL6A2	P12110	G3QM74	A0A2J8RBT8	A0A2J8M8T3
COL7A1	Q02388	XP_055238310	A0A2J8TG09	H2QMJ5
COL9A1	P20849	G3R409	H2PJI8	A0A6D2Y0Y9
COL10A1	Q03692	G3S3J2	A0A6D2VST1	H2QTL5
COL11A1	P12107	A0A2I2Z7V8	A0A2J8VCH4	A0A6D2W5P1
COL11A2	P13942	G3R2X9	H2PL45	H2R4E0
COL18A1	P39060	XP_055229711.1	A0A2J8RBR0	H2RAH2
COL21A1	Q96P44	XP_055246310.1	A0A2J8SSP6	A0A2J8P166
COL22A1	Q8NFW1	G3R3K3	H2PR96	A0A2J8LF91
COL23A1	Q86Y22	G3QVJ2	H2PHJ9	A0A2J8JTU5
COL24A1	Q17RW2	G3QDU7	H2N6W1	A0A2I3RU50
COL27A1	Q8IZC6	G3RVE3	H2PT57	H2QXR2

Section 5. Skeletal element profiles, faunal taphonomy, and hominin behaviours

5.1. Skeletal element profiles

At BKC, 1,910 of 2,005 identifiable specimens (Supplementary Dataset 5C) have been classified into specific bone types. Of these, 1,716 specimens are from layers 2-7 and 10-11. For each selected layer, there is a wide skeletal element representation (SI Fig. 5.1). As a whole, front/hind limbs (1,041/1,716), including the scapula, humerus, radius-ulna, femur, tibia, metacarpus and metatarsals, account for more than 50% of the total NISP in each layer. Elements related to the head bones (skull, including teeth, and horn/antler, 245/1,716, 14.28%) and axial bones (vertebrae, rib and pelvis, 266/1,716, 15.50%) are present in all layers. Phalanges (100/1,716) and carpals/tarsals (64/1,716), are only absent in layer 7.

Notably, bone types of single taxa varied across layers, except for Caprinae (SI Fig. 5.2). The distribution of Caprinae shows that hominins did not transport carcasses to the cave selectively, but instead brought the entire carcasses into BKC. In addition, the presence of a complete butchery carcass processing of Caprinae (Fig. 1e), which also provides extra evidence of human activities (see Supplementary Information Section 5.3 for details). It is therefore reasonable to assume that foraging activities took place nearby.

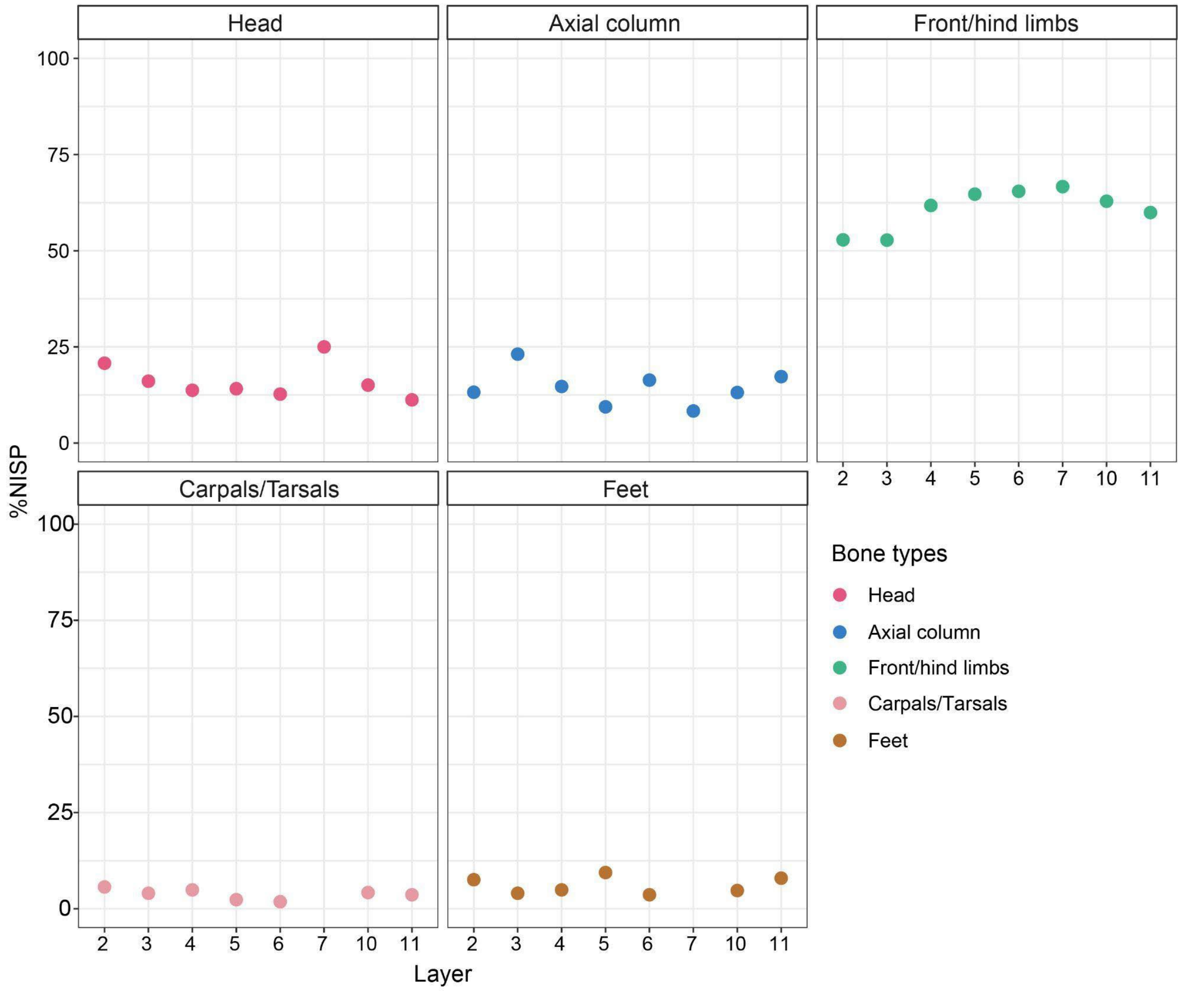

SI Fig. 5.1. The %NISP of different bone types in each stratigraphic unit. NISP are based on Supplementary Dataset 5C.

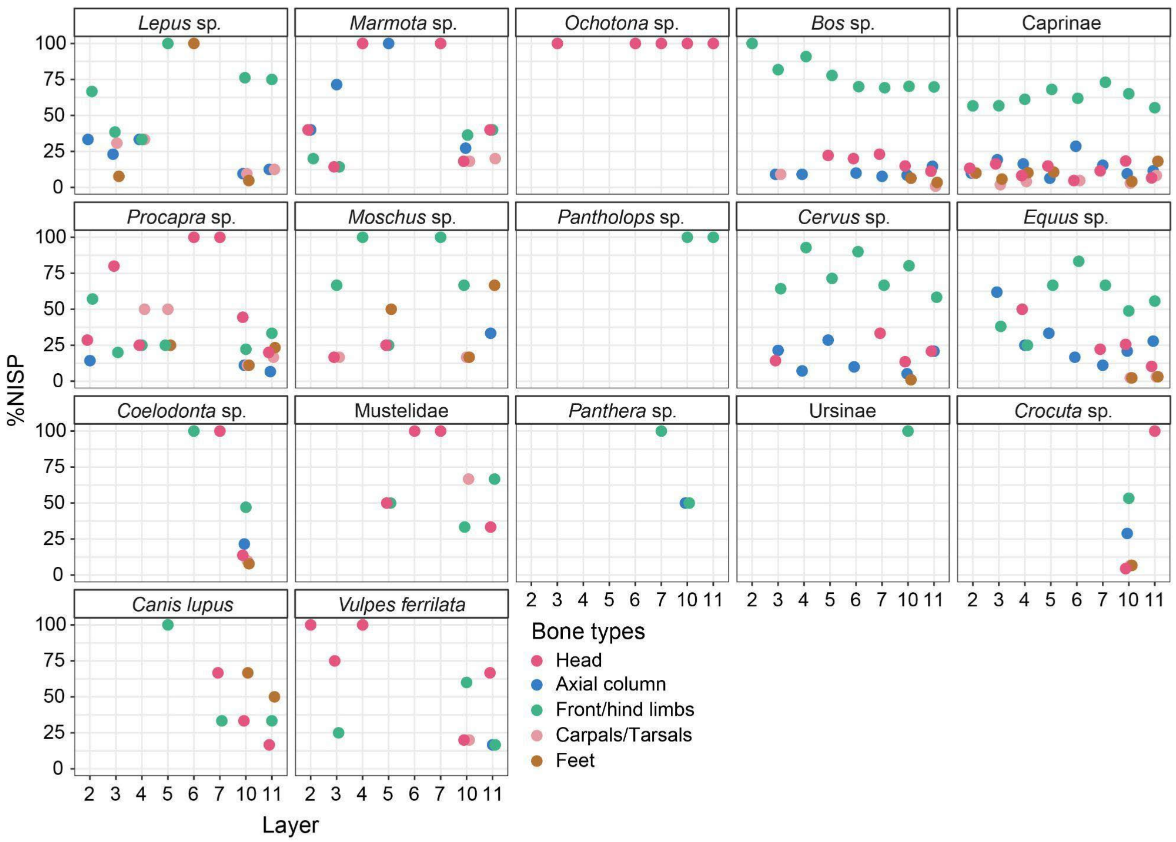

SI Fig. 5.2. The %NISP of bone types of selected taxonomic groups identified by both morphological and ZooMS analysis in each stratigraphic unit. Selected taxa and NISP are based on Extended Data Table 1.

5.2. Faunal taphonomy

In general, hominins, carnivores, raptors, rodents, and fluvial transportation could all facilitate the accumulation of bones at archeological sites[41]. Usually raptors mainly prey on rodents, rabbits, birds and small primates[108] and tend to leave digestive erosion traces on the bones. Taxonomic composition at BKC is dominated by large and medium-sized herbivores (Supplementary Dataset 5), thus obviously different from bone assemblages accumulated by raptors. In addition, there is a lack of evidence of digestive erosion by stomach acid on the bone surfaces at BKC. Therefore, we can exclude the possibility of raptor accumulation of bones at BKC. The lack of water-abrasion marks on the bone surfaces demonstrates that the BKC faunal assemblage was not formed by hydraulic transportation either. In addition, bone sizes range

from smaller than 20 mm to larger than 100 mm (SI Fig. 4.1a), showing little sorting or winnowing processes, providing further evidence of minimal fluvial disturbance.

Three types of biogenic modifications (rodent, carnivore and anthropogenic) are present in the BKC faunal assemblage. The proportion of rodent and carnivore modifications is extremely low (SI Table 5.1). Rodent modifications, represented by incisor gnawing traces, are present on just three specimens (0.1% of the 2,005 taxonomically identified specimens, from layers 1, 4 and 11), two of which are identified on *Lepus* sp. specimens and one on a *V. ferrilata* specimen (SI Table 5.1). This percentage is much lower than what is observed in assemblages from common rodent (porcupine) lairs (>22%)[38]. Thus, we exclude rodents as the major agents of bone accumulation. In addition, the animal bones accumulated by rodents are usually heavily weathered[41]. However, weathering of the identified specimens at BKC is relatively low, with 88.74% of the specimens (*n* = 1,616 out of *n* = 1,821) assigned to Behrensmeyer[39] weathering stages I or II. Carnivore modifications are slightly more common than rodent modifications, but still very rare (*n* = 16, 0.79% of the *n* = 2,005 taxonomically identified specimens, present in layers 1, 2, 4, 7, 10 and 11), which is far lower than the experimental percentage observed when carnivores have primary access to animal carcasses[109,110]. Such traces are present on specimens belonging to a wide range of animal body-size categories (small, medium and large; SI Table 5.1). However, no carnivore coprolites were found during the excavation. Therefore, the current evidence suggests that carnivores are unlikely to be the primary accumulators of the BKC faunal assemblage.

In contrast to the low presence of rodent and carnivore modifications, a significant proportion of the skeletal assemblage (*n* = 545) contains one or several anthropogenic modifications (Extended Data Fig. 4a and 5). Of those bones with anthropogenic modifications, 386 specimens could be identified to specific taxonomic groups (SI Table 5.2), representing about 19.25% of the total NISP (*n* = 2,005). Anthropogenic modifications include cut marks (*n*

= 282), anvil marks (n = 5), and percussion notches (n = 175). It is noteworthy that 57.30% (51/89) specimens from layers 8-9 contain one or several anthropogenic modifications, while only seven of them could be taxonomically identified (*Bos* sp., Caprinae, Cervinae/*Gazella* sp., *Marmota* sp., V. *vulpes*/*ferrilata*; Supplementary Dataset 5C). The %NISP of Caprinae specimens with anthropogenic modifications is around 25%, with no significant differences across layers (layers 2-7 and 10-11, SI Fig. 5.3). Anthropogenic modifications on Cervinae/*Gazella* sp. and *Bos* sp. specimens are present in most layers, except for layer 2. Specimens of other taxonomic groups with anthropogenic modifications, i.e., *Coelodonta* sp., *Pantholops* sp., *Procapra* sp., and *Panthera* sp., are also present across the stratigraphy (SI Fig. 5.3). In particular, anthropogenic modifications such as cut marks and percussion notches are also present on some large carnivores, like spotted hyena and wolf specimens (SI Table 5.2 and Fig. 2). Burned bones are also present at BKC (Extended Data Fig. 5m), though on only six specimens from layers 5, 7 and 8. Based on this limited number of burned bones, it is currently difficult to tell whether the burning was anthropogenic or natural in origin. Given the depositional environment and the high anthropogenic input overall, we deem the latter less likely. In addition, a high proportion of hominin-modified bones co-occur with lithic artefacts in each layer[4]. Based on the above analysis, we conclude that the BKC faunal assemblage accumulated to a large extent due to hominin activities.

SI Table 5.1. Distribution of identifiable bone specimens with carnivore or rodent modifications across stratigraphic units. Taxa and NISP are based on Supplementary Dataset 5C. Absent data indicates that no specimens of a certain taxonomic group with anthropogenic modifications were observed in a particular layer.

**Modification traces**	**Taxon**	**Layer**						**Total NISP**
		**1**	**2**	**4**	**7**	**10**	**11**	
**Carnivore**	Aves	1					1	**2**
	Bos sp.	1				2		**3**
	Caprinae	1	1	1	1	1		**5**
	Crocuta sp.					1		**1**
	Equidae				1			**1**
	Leporidae						1	**1**
	Marmota sp.	1						**1**
	Procapra sp.			1			1	**2**
	**Total NISP**	**4**	**1**	**2**	**2**	**4**	**3**	**16**
**Rodent**	Leporidae	1						1
	Vulpes vulpes/ferrilata						1	1
	**Total NISP**	**1**					**1**	**2**

SI Table 5.2. Distribution of identifiable bone specimens with anthropogenic modifications across stratigraphic units. Taxa and NISP are based on Supplementary Dataset 5C. Absent data indicates that no specimens of a certain taxonomic group with anthropogenic modifications were observed in a particular layer.

**Taxon**	**Layer**													**Total NISP**
	**1**	**2**	**3**	**4**	**5**	**6**	**7**	**8**	**9**	**10**	**11**	**Pits (H1 and H3)**	**Unknown**	
**Aves**				1										**1**
***Bos* sp.**	4		2	3	1	2	2	1	1	9	12	11		**48**
**Bovidae**											6			**6**
**Canidae (not *Vulpes vulpes/ferrilata*)**										1	1			**2**
**Caprinae**	5	8	34	16	13	5	11	1	1	42	29	18	1	**184**
**Cervinae/*Gazella* sp.**			2	8	2	1	3		1	17	7	6	1	**48**
***Crocuta* sp.**										4				**4**
**Equidae**			3				3			7	20	2		**35**
**Equidae/Cervinae/Antilopinae**											1			**1**
**Leporidae**	1											1		**2**
***Marmota* sp.**									1					**1**
**Moschidae**				1	1									**2**
**Pantherinae**							1							**1**
***Pantholops* sp.**										2				**2**
**Pecora**			1							8	27	1		**37**
***Procapra* sp.**		3								1	1	4		**9**
**Rhinocerotidae**										2				**2**
Vulpes vulpes/ferrilata								1						**1**
**Total NISP**	**10**	**11**	**42**	**29**	**17**	**8**	**20**	**3**	**4**	**93**	**104**	**43**	**2**	**386**

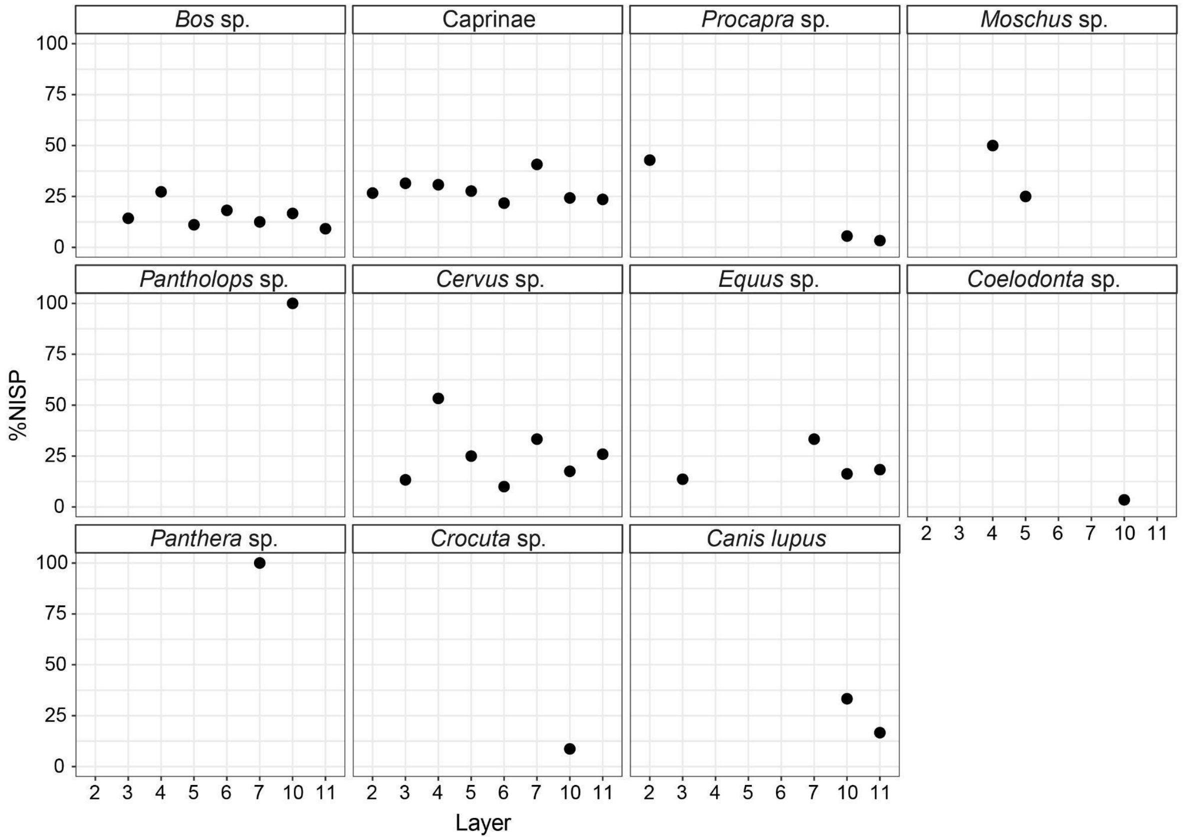

SI Fig. 5.3. The percentage of anthropogenic modifications present on specimens of different taxonomic groups across stratigraphic units. Taxa and NISP are based on Extended Data Table 1. Absent data indicates that no specimens of a certain taxonomic group with anthropogenic modifications were observed in a particular layer.

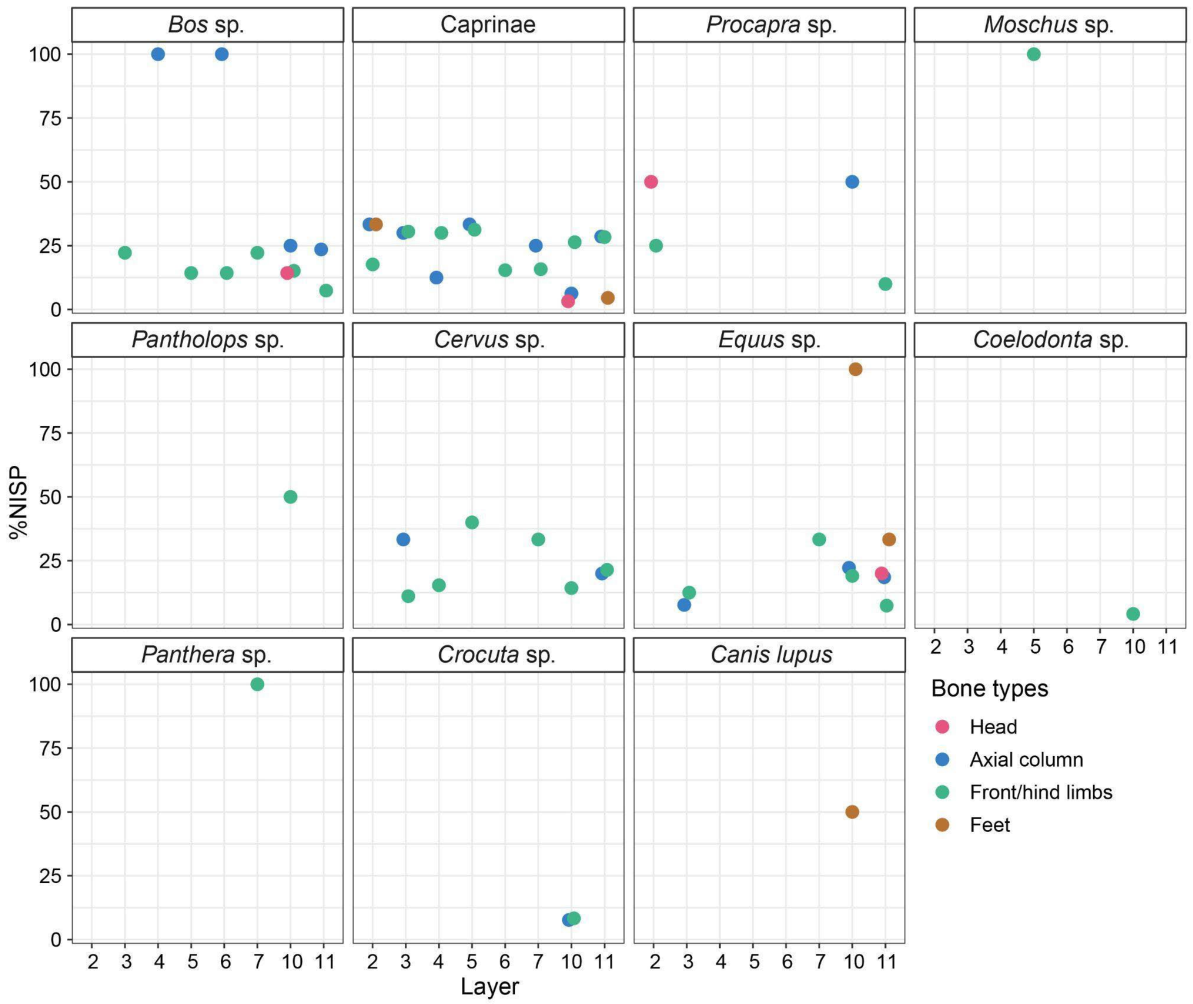

SI Fig. 5.4. The distribution of cut marks present on the specific bone types (head, axial column, front/hind limbs and feet) of different taxonomic groups across stratigraphic units. Taxa and NISP are based on Extended Data Table 1. Absent data indicates that no specimens of a certain taxonomic group with anthropogenic modifications were observed in a particular layer.

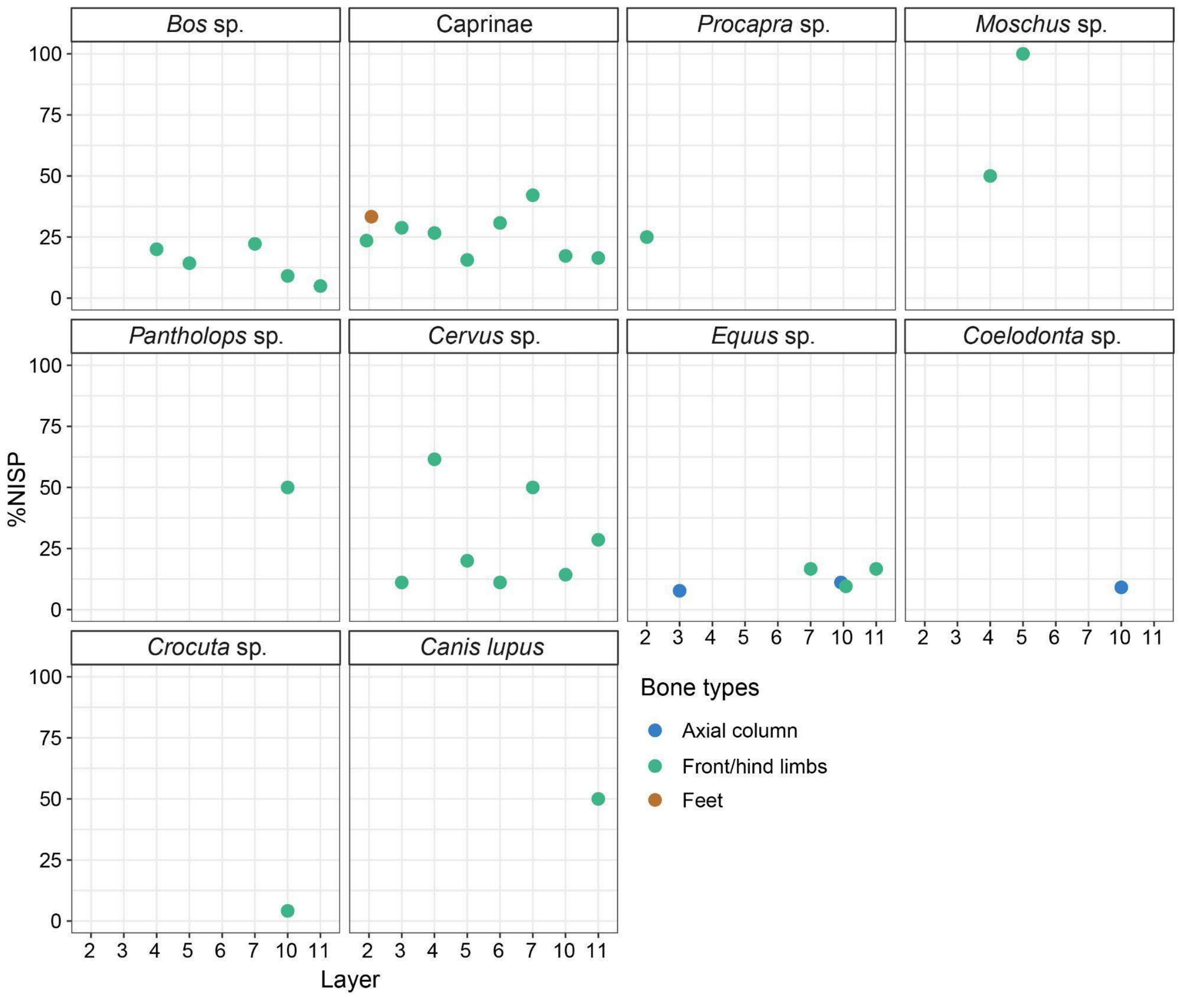

SI Fig. 5.5. The distribution of percussion notches present on the specific bone types (head, axial column, front/hind limbs and feet) of different taxonomic groups across stratigraphic units. Taxa and NISP are based on Extended Data Table 1. Absent data indicates that no specimens of a certain taxonomic group with anthropogenic modifications were observed in a particular layer.

5.3. Processing of animal carcasses by hominins

As one of important resources for hominins, animals not only provide food resources (e.g. meat, fat, and marrow), but also non-food materials (e.g. hide and bone tool blanks)[111,112]. When hominins try to obtain these resources, corresponding anthropogenic marks are usually left on the bone surfaces, such as cut marks, percussion notches, and so on[40,113]. These bone surface modifications and other associated evidence of butchering and bone tool manufacturing are frequently used for reconstructing the *chaîne opératoire* of carcass processing in hominin groups[114-117].

It is worth noting that the majority of cut marks observed in the BKC faunal assemblage are concentrated on limb elements (SI Fig. 5.4, Extended Data Fig. 5c, 5d and 5g to 5i), especially the meat-rich upper/intermediate limb shaft parts rather than the almost meat-free lower limb shaft parts (SI Table 5.3). These elements are generally difficult to taxonomically identify based on morphological characteristics. However, cut marks related to oblique and longitudinal incisions on the shaft of the limb bones are usually treated as associated with filleting of large muscles[118-120], indicating the primary access to the carcasses of these animals by hominins[121]. Fortunately, by integrating the ZooMS approach, the majority of these limb shaft fragments were successfully identified taxonomically, indicating filleting activities throughout the BKC stratigraphy (SI Fig. 5.6). Dismembering and defleshing activities, which are usually performed simultaneously[40,122], might have also existed at BKC, as indicated by cut marks on the articulations of front/hind limb bones of *P. nayaur* (Extended Data Fig. 5c and 5d). Similar butchery behaviours have also been observed for other Pleistocene hominins, like Neanderthals[111,123], and other archaic hominins in China, e.g. Xuchang[124,125] and Xujiayao[126].

Cut marks on phalanges and metapodials closely resemble those left on bones during experimental skinning[39,113,127]. Cut marks on phalanges or metapodials of Caprinae, *Cervus* sp. and *Equus* sp. in our data (Extended Data Fig. 5e and 5f and SI Fig. 5.6), are probably also

associated with skinning activities, especially as furs and skins of these taxa are commonly used for clothing-making in the past and today[128]. In particular, the procurement and preparation of animal hides, for example in the context of making clothing, could have been highly relevant for hominins to survive in cold environments. Thus, the evidence for skinning activities at BKC might have helped Denisovans to adapt to the cold, high-altitude TP.

In addition, a total of 733 identifiable front/hind limb shaft specimens were assessed for breakage patterns, including dry breakage (n = 27), green/fresh breakage (n = 743), and intermediate breakage types (n = 3). The dominance of green/fresh bone breakage, together with the high frequencies of percussion notches (SI Fig. 5.5 and Extended Data Fig. 5i and 5j) and the presence of bone flakes (Extended Data Fig. 5k and 5l) suggest marrow extraction activities at BKC.

The above hominin behaviours, including dismembering, filleting, skinning and marrow extraction, are present in almost all layers of BKC (SI Fig. 5.6), suggesting that butchering activities at BKC involved a complete sequence of animal carcass processing, especially herbivorous.

Informal bone tools have been recorded in many Early and Middle Paleolithic assemblages[129-131]. Formal bone tools in the Upper Paleolithic period were very common and usually deliberately manufactured with a particular function, such as projectile points, needles, awls, etc. Compared to formal bone tools, informal bone tools are generally not formally shaped into a specific shape.

At BKC, four informal bone or tooth tools (one possible tooth retoucher and three expedient bone tools) were recovered from layers 4, 9, 10 and 11 (Fig. 2d and 2e, Extended Data Table 2), suggesting that animal carcasses also constituted important raw materials in the technological system of the BKC hominin groups. The possible retoucher is derived from an *Equus* sp. right lower P2. The buccal surface of the tooth bears two contiguous, nearly

triangular and deep pits with V-shaped profile, which are likely due to resharpening lithic tools. Retouchers are the most common bone tools in Middle Paleolithic sites[131-134], and are usually used as intermediaries to shape and/or refine stone tools by percussion or pressure flaking during the lithic *chaîne opératoire*. Usually, retouchers are made of limb bone diaphyses in the Middle and Upper Paleolithic periods, however, tooth retouchers are also known[135,136]. However, for this tooth specimen from BKC, the marks on the buccal side could also be due to marrow extraction from the mandible in which the tooth was embedded. Expedient bone tools, usually represented as a diaphysis fragment with flake scars on the cortical and/or medullary surfaces on the edge formed during knapping. Direct percussion of the bone for (re)shaping tools, such as bone handaxes or bone bifaces, appeared as early and widespread as stone tools[130,137]. The three expedient bone tools at BKC are also made of diaphysis fragments and provide additional evidence for bone tool manufacturing among Denisovans[138].

In conclusion, the *chaîne opératoire* of butchery and carcass processing described here, including systematic butchery (dismembering, filleting, skinning and bone marrow extraction) and bone material recycling (i.e. the manufacture of informal bone tools), suggests that Denisovans had a similar capacity to acquire and utilise animal resources as other Middle and Late Pleistocene hominin groups, such as Neanderthals in western Eurasia, other archaic hominins in eastern Eurasia, and modern humans in Africa (and possibly beyond)[49,131]. Further research on Denisovan faunal assemblages across Eastern Eurasia, in comparison to the existing data from BKC, Denisova Cave, and Tam Ngu Hao 2 (Cobra Cave), will therefore provide the opportunity to derive spatiotemporal insights into Denisovan animal resource use.

SI Table 5.3. The number and percentages of limb shaft specimens with cut marks by element and body parts from BKC. These data do not include small-sized animals and birds. It's worth noting that most of the limb shaft specimens with cut marks cannot be identified as skeletal elements or limb parts, as they are very fragmented and lack morphological characteristics. However, intermediate limb shaft specimens are taller than the upper limb shafts, probably because the unique anatomical characteristics of the radius-ulnar and tibial diaphysis make them easier to identify.

Limb parts	N	%
Humerus	8	3.3
Femur	5	2
Indeterminate upper limbs	8	3.3
**Total upper limbs**	**21**	**8.6**
Radius-Ulna	20	8.1
Tibia	43	17.4
**Total intermediate limbs**	**63**	**25.5**
Indeterminate upper/intermediate limbs	98	39.8
**Total upper/intermediate limbs**	**182**	**73.9**
Metapodials	26	10.6
**Total lower limbs**	**26**	**10.6**
Indeterminate limbs	38	15.5
**Total**	**246**	**100**

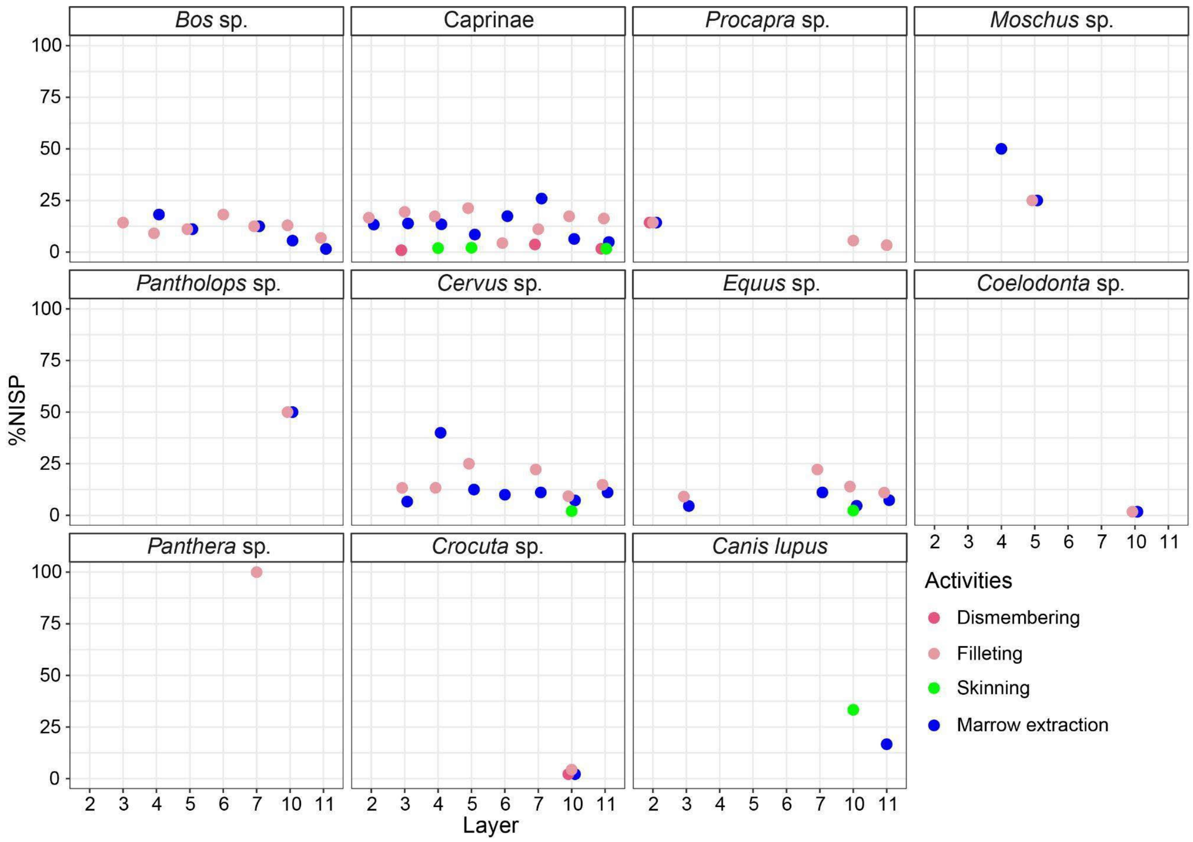

SI Fig. 5.6. The percentage of identifiable specimens reflecting hominin butchering and consumption activities (dismembering, filleting, skinning and marrow extraction) in each stratigraphic unit. Taxa and NISP are based on Extended Data Table 1.

Section 6. Deamidation

At BKC, we are able to obtain collagen deamidation values for a total of 1,101 specimens. For specimens extracted separately using acid and ambic extraction protocols, we observe comparable deamidation values (Extended Data Fig. 7). A limited duration acid demineralization of 1 to 2 days has therefore had a negligible impact on the deamidation results. The high consistency of deamidation values between T2 and T3 in the same stratum (SI Fig. 6.2) further confirms the shared stratigraphic sequence of T2 and T3 and allows us to consider the deamidation data as a single sample set. Therefore, in the following data analysis, we only focus on the deamidation values from the acid-extracted collagen and analyse the deamidation values by combining samples from the same stratum of T2 and T3.

Deamidation levels of COL1α1 508-519 and COL1α1 435-453 between layers show a similar pattern (SI Fig. 6.1). These range from non-deamidated deamidation levels (layer 1 and some samples from two historical pits), to values close to zero in layers 10 and 11, where glutamines are almost completely converted to glutamic acids. One specimen classified into layer 2 based on depth has a deamidation value as 1, is significantly different from other specimens from layer 2, all of which have deamidation values under 0.8 and are close to the specimens radiocarbon dated to around ~30 ka (Supplementary Dataset 2). By rechecking the 3D coordinates for this sample, we found this sample is located near the boundary of the historical pit H1 and layer 2. Radiocarbon dating results show that this specimen is dated to ~740 cal BP (Supplementary Dataset 2), consistent with the age of H1. This shows the potential for detecting outliers based on deamidation values, especially historical or modern intrusions into prehistoric assemblages.

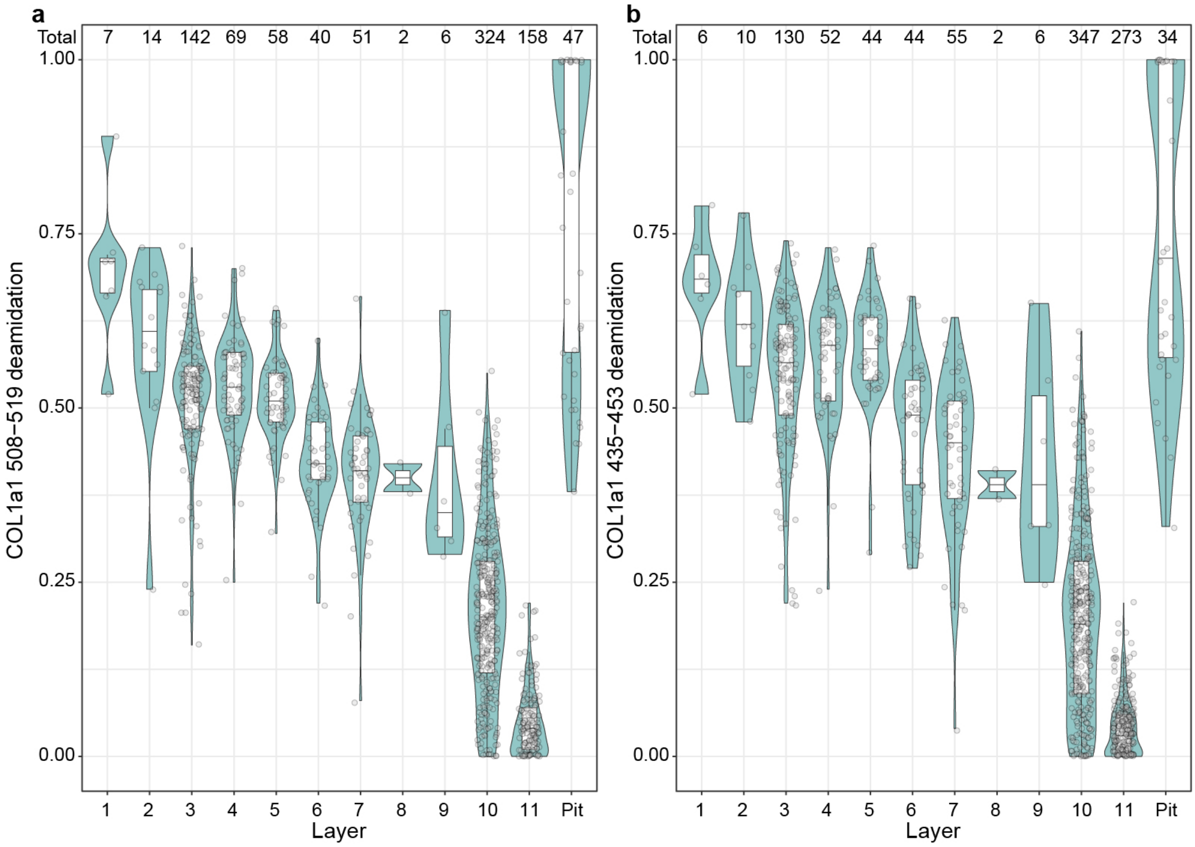

SI Fig. 6.1. Box and violin plots of the deamidation values of COL1α1 508-519 (a) and COL1α1 435-453 (b) per stratigraphic unit. The final violin gives the deamidation values of bone specimens from the two historical pits (H2 and H3). Here, the value of 1 indicates no deamidation while the value of 0 indicates complete deamidation of the glutamine in the marker peptide COL1α1 508-519. The total number of data points obtained for each layer is given at the top. The box plots within the violin plots contain the range of the data with whiskers extending to 1.5 times the interquartile range. The boxes indicate the upper and lower quartiles, while the centre line indicates the median. All individual data points are represented by overlaid dot plots, and any data points outside the range of the box plot can be considered outliers.

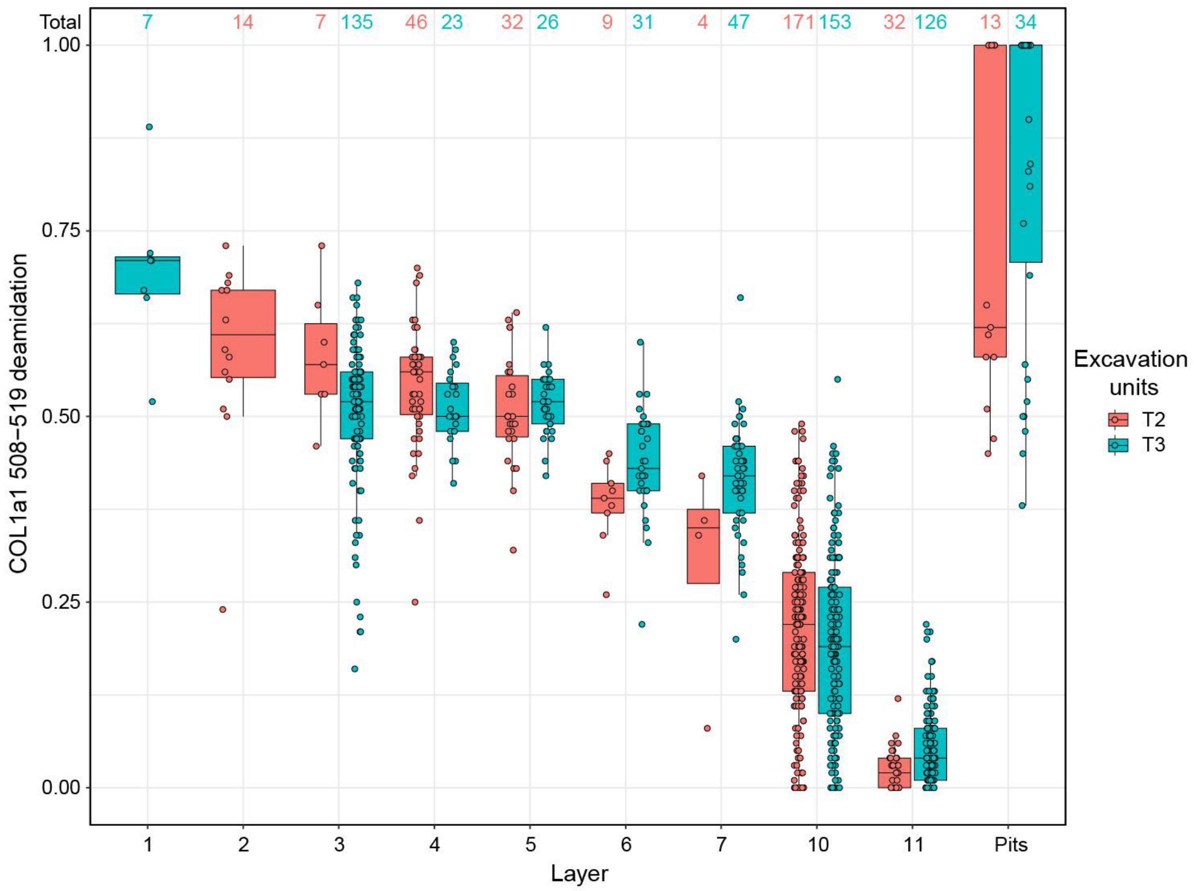

SI Fig. 6.2. Box plots of COL1α1 508-519 deamidation values of specimens from T2 or T3 per in each layer. Here, the value of 1 indicates no deamidation while the value of 0 indicates complete deamidation of the glutamine in the marker peptide COL1α1 508-519. The total number of data points analysed for each layer of T2 or T3 is given at the top. The box plots contain the range of the data with whiskers extending to 1.5 times the interquartile range. The boxes indicate the upper and lower quartiles, while the centre line indicates the median. All individual data points are represented by overlaid dot plots, and any data points outside the range of the box plot can be considered outliers.

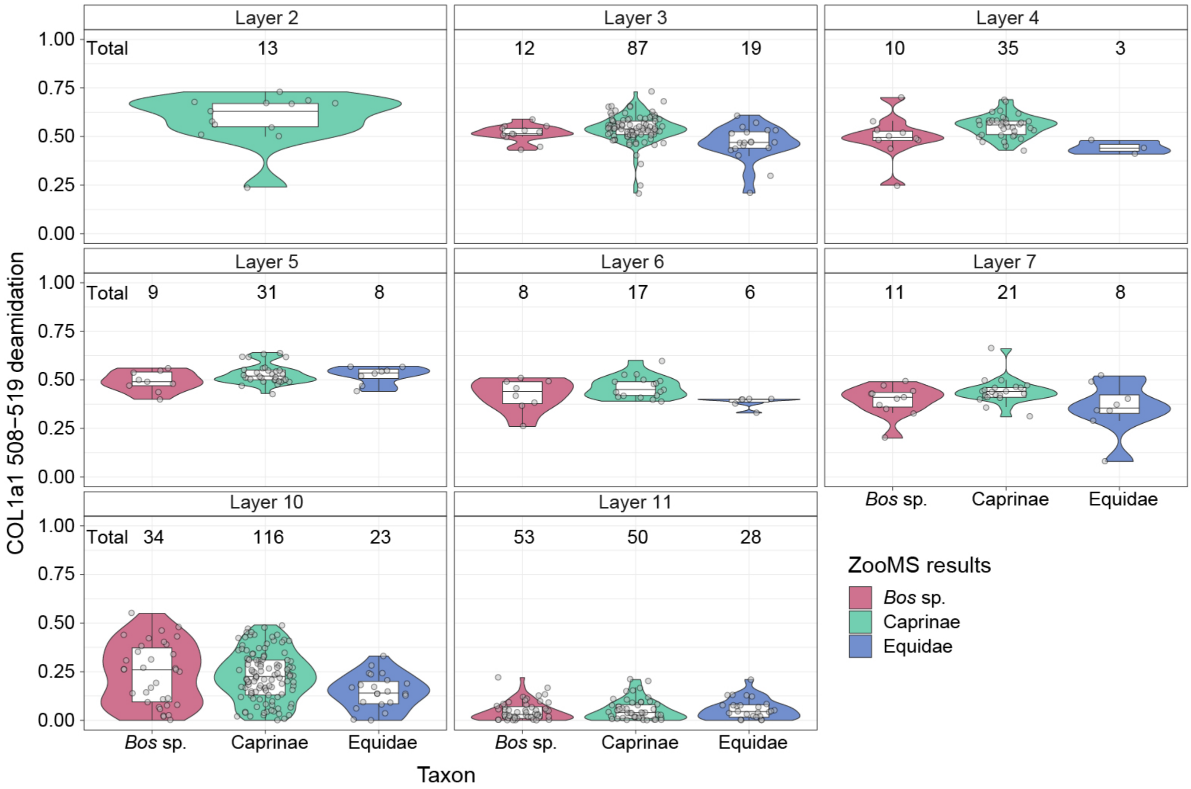

SI Fig. 6.3. Violin plots of COL1α1 508-519 deamidation values of the dominant taxonomic groups in each layer. Here, the value of 1 indicates no deamidation while the value of 0 indicates complete deamidation of the glutamine in the marker peptide COL1α1 508-519. The box plots within the violin plots contain the range of the data with whiskers extending to 1.5 times the interquartile range. The boxes indicate the upper and lower quartiles, while the centre line indicates the median. All individual data points are represented by overlaid dot plots, and any data points outside the range of the box plot data can be considered outliers. The total number of data points analysed for each taxonomic group in each layer is presented in the figure.

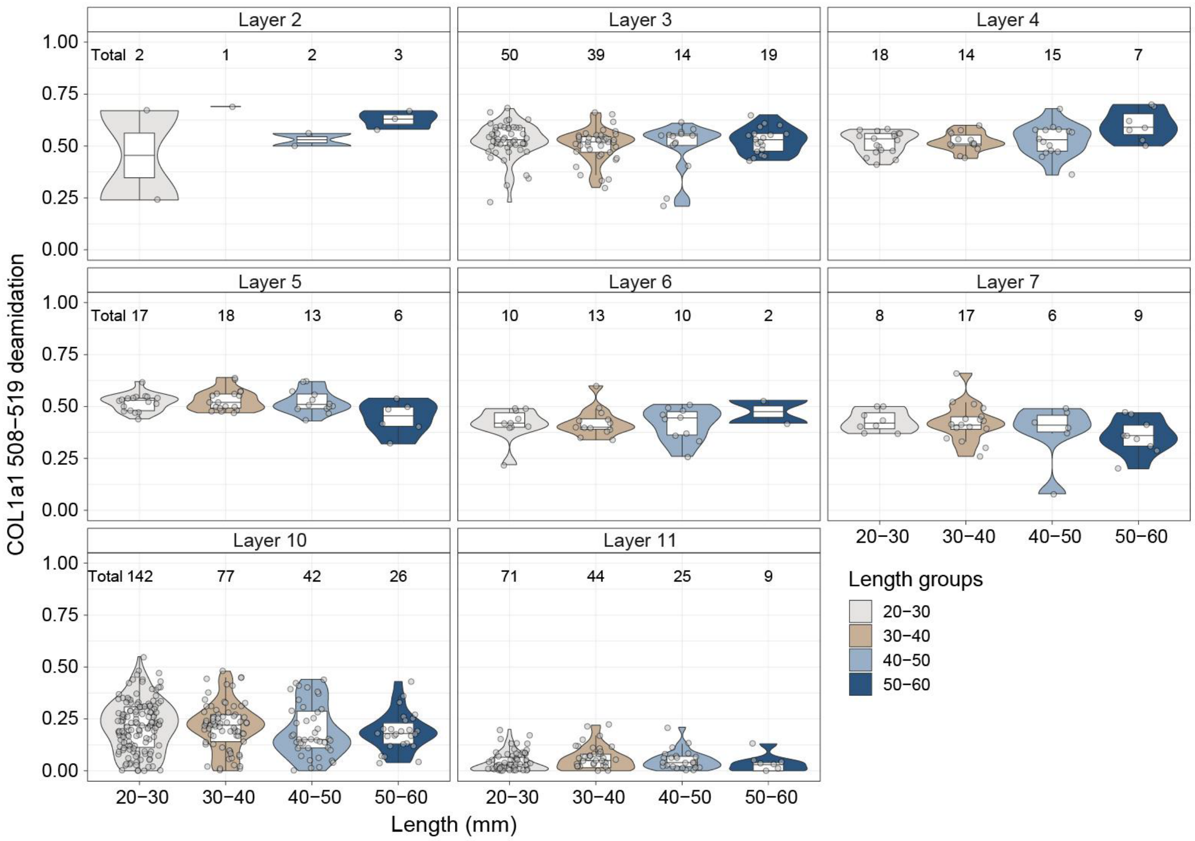

SI Fig. 6.4. Violin plots of COL1α1 508-519 deamidation values of different bone length groups in each layer. Here, the value of 1 indicates no deamidation while the value of 0 indicates complete deamidation of the glutamine in the marker peptide COL1α1 508-519. The box plots within the violin plots contain the range of the data with whiskers extending to 1.5 times the interquartile range. The boxes indicate the upper and lower quartiles, while the centre line indicates the median. All individual data points are represented by overlaid dot plots, and any data points outside the range of the box plot data can be considered outliers. The total number of data points analysed for each length group in each layer is presented in the figure.

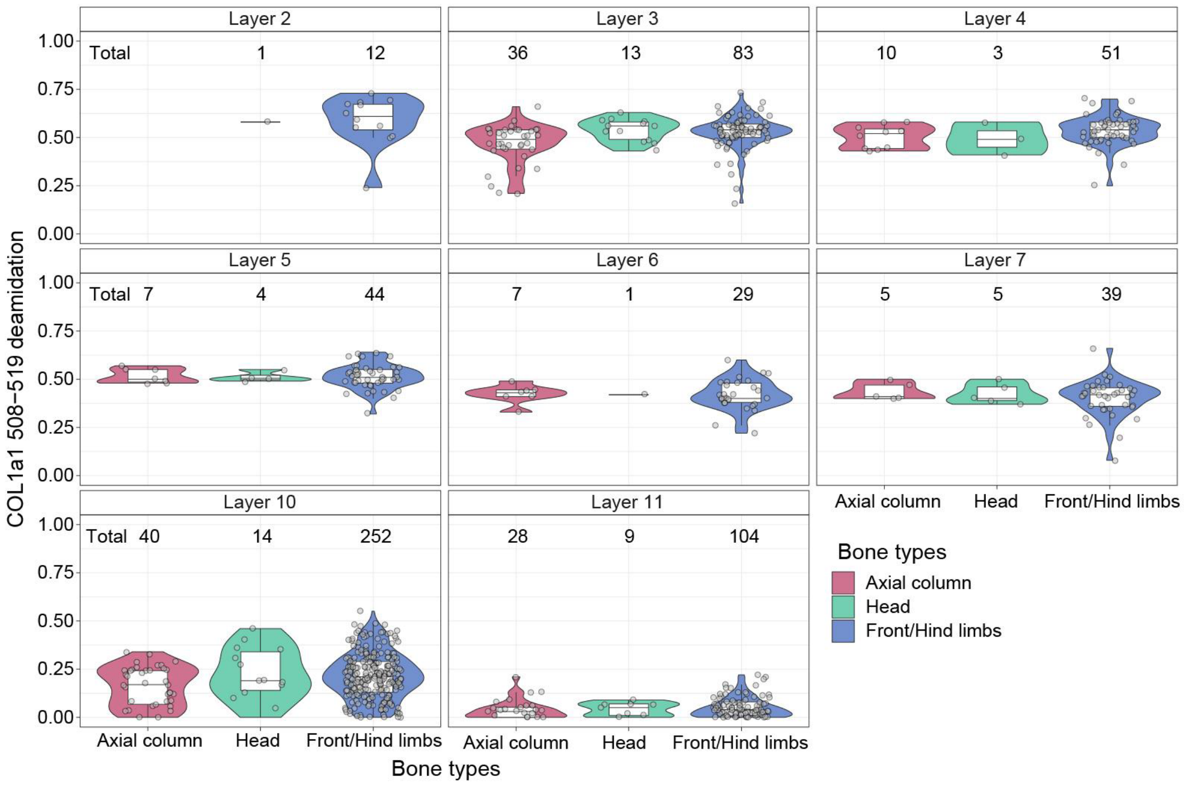

SI Fig. 6.5. Violin plots of COL1α1 508-519 deamidation values of different bone types (front/hind limbs, cranial, axial bones) in each layer. Here, the value of 1 indicates no deamidation while the value of 0 indicates complete deamidation of the glutamine in the marker peptide COL1α1 508-519. The box plots within the violin plots contain the range of the data with whiskers extending to 1.5 times the interquartile range. The boxes indicate the upper and lower quartiles, while the centre line indicates the median. All individual data points are represented by overlaid dot plots, and any data points outside the range of the box plot data can be considered outliers. The total number of data points analysed for each bone type in each layer is presented in the figure.

Section 7. Possible subdivisions of layer 10

During our ZooMS and morphological analysis, we observed some significant differences in taxonomic composition between the sublayers of layer 10 (SI Fig. 7.1a). For example, some small size animals, such as marmots and birds, are absent in layer 10b, but present in layers 10a, 10c and 10d. Subsequent analysis also revealed that the Shannon index obtained for layer 10b is different from other layers 10a, 10c and 10d (SI Fig. 7.1b). Likewise, although average deamidation levels of the four sublayers all lie between the average values of layers 9 and 11, the deamidation distributions differ among the sublayers as well (SI Fig. 7.1c), especially for layer 10a. Based on these observations, future chronological analysis of these sublayers is important to understand the formation process of these sublayers, for example assessing whether they are the result of rapid deposition processes or complex taphonomic site formation processes.

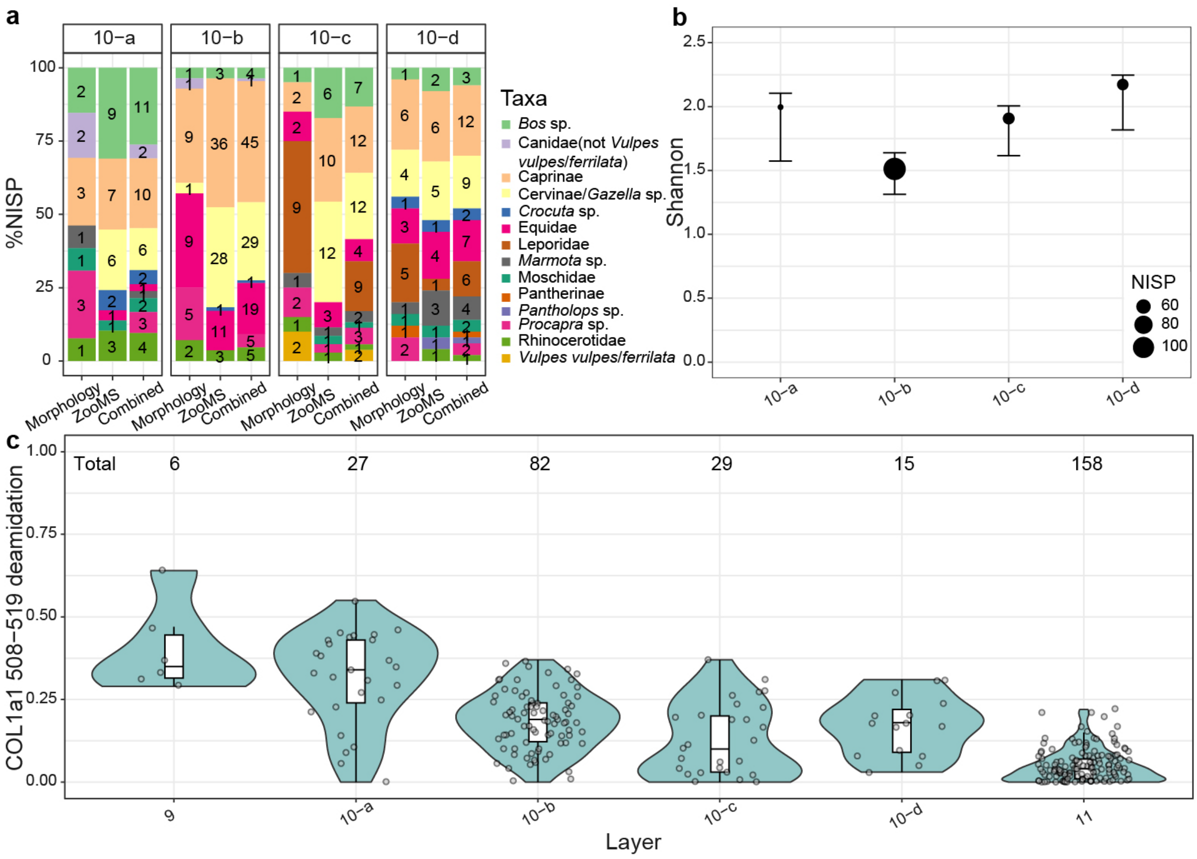

SI Fig. 7.1. Analysis of layer 10 and its possible subdivisions. Taxonomic composition established by morphology, ZooMS and combined (**a**), and Shannon index (**b**) of layers 10a, 10b, 10c, and 10d, and COL1α1 508-519 deamidation values (**c**) of layers 9, 10a, 10b, 10c, 10d and 11. For (**a**), the NISP counts of each taxon are shown on their respective bar segments. For (**c**), the value of 1 indicates no deamidation while the value of 0 indicates complete deamidation of the glutamine in the marker peptide COL1α1 508-519. The box plots within the violin plots contain the range of the data with whiskers extending to 1.5 times the interquartile range. The boxes indicate the upper and lower quartiles, while the centre line indicates the median. All individual data points are represented by overlaid dot plots, and any data points outside the range of the box plot data can be considered outliers. The total number of data points analysed for each layer is presented in the figure.

References

73 Zhang, D. et al. Earliest parietal art: hominin hand and foot traces from the middle Pleistocene of Tibet. *Sci. Bull.* **66**, 2506-2515 (2021).

74 Zheng, Z. et al. Palaeolithic site of Piluo in Daocheng County, Sichuan. *Archaeology* **7**, 723-734 (2022).

75 Zhang, X. L. et al. The earliest human occupation of the high-altitude Tibetan Plateau 40 thousand to 30 thousand years ago. *Science* **362**, 1049-1051 (2018).

76 Guan, Y. et al. Microblade remains from the Xishahe site, North China and their implications for the origin of microblade technology in Northeast Asia. *Quat. Int.* **535**, 38-47 (2020).

77 Zhang, D. et al. History and possible mechanisms of prehistoric human migration to the Tibetan Plateau. *Sci. China Earth Sci.* **59**, 1765-1778 (2016).

78 Madsen, D. B. et al. The Late Upper Paleolithic occupation of the northern Tibetan Plateau margin. *J. Archaeol. Sci.* **33**, 1433-1444 (2006).

79 Ni, X. et al. Massive cranium from Harbin in northeastern China establishes a new Middle Pleistocene human lineage. *Innovation* **2**, 100130 (2021).

80 Buckley, M., Collins, M., Thomas-Oates, J. & Wilson, J. C. Species identification by analysis of bone collagen using matrix-assisted laser desorption/ionisation time-of-flight mass spectrometry. *Rapid Commun. Mass Spectrom.* **23**, 3843-3854 (2009).

81 Buckley, M. et al. Species identification of archaeological marine mammals using collagen fingerprinting. *J. Archaeol. Sci.* **41**, 631-641 (2014).

82 Janzen, A. et al. Distinguishing African bovids using Zooarchaeology by Mass Spectrometry (ZooMS): new peptide markers and insights into Iron Age economies in Zambia. *PLOS ONE* **16**, e0251061 (2021).

83 Paladugu, R. et al. Your horse is a donkey! Identifying domesticated equids from Western Iberia using collagen fingerprinting. *J. Archaeol. Sci.* **149**, 105696 (2023).

84 Peters, C. et al. Species identification of Australian marsupials using collagen fingerprinting. *Royal Soc. Open Sci.* **8**, 211229 (2021).

85 Rüther, P. L. et al. SPIN enables high throughput species identification of archaeological bone by proteomics. *Nat. Commun.* **13**, 2458 (2022).

86 Welker, F. et al. Using ZooMS to identify fragmentary bone from the Late Middle/Early Upper Palaeolithic sequence of Les Cottés, France. *J. Archaeol. Sci.* **54**, 279-286 (2015).

87 Brown, S. et al. Identification of a new hominin bone from Denisova Cave, Siberia using collagen fingerprinting and mitochondrial DNA analysis. *Sci. Rep.* **6**, 23559 (2016).

88 Hublin, J.-J. et al. Initial Upper Palaeolithic *Homo sapiens* from Bacho Kiro Cave, Bulgaria. *Nature* **581**, 299-302 (2020).

89 Lanigan, L. T. et al. Multi-protease analysis of Pleistocene bone proteomes. *J. Proteomics* **228**, 103889 (2020).

90 Ruebens, K. et al. Neanderthal subsistence, taphonomy and chronology at Salzgitter-Lebenstedt (Germany): a multifaceted analysis of morphologically unidentifiable bone. *J. Quat. Sci.* **38**, 471-487 (2023).

91 Bouchard, G. P., Riel-Salvatore, J., Negrino, F. & Buckley, M. Archaeozoological, taphonomic and ZooMS insights into The Protoaurignacian faunal record from Riparo Bombrini. *Quat. Int.* **551**, 243-263 (2020).

92 Mylopotamitaki, D. et al. *Homo sapiens* reached the higher latitudes of Europe by 45,000 years ago. *Nature* **626**, 341-346 (2024).

93 Jacobs, Z. et al. Timing of archaic hominin occupation of Denisova Cave in southern Siberia. *Nature* **565**, 594-599 (2019).

94 Bacon, A.-M. et al. Palaeoenvironments and hominin evolutionary dynamics in southeast Asia. *Sci. Rep.* **13**, 16165 (2023).

95 Le Meillour, L. et al. Increasing sustainability in palaeoproteomics by optimizing digestion times for large-scale archaeological bone analyses. *iScience* **27**, 109432 (2024).

96 Hou, X. et al. Paleogenomes reveal a complex evolutionary history of Late Pleistocene bison in northeastern China. *Genes* **13**, 1684 (2022).

97 Campana, M. G., Robinson, T., Campos, P. F. & Tuross, N. Independent confirmation of a diagnostic sheep/goat peptide sequence through DNA analysis and further exploration of its taxonomic utility within the Bovidae. *J. Archaeol. Sci.* **40**, 1421-1424 (2013).

98 Wang, X. & Hoffmann, R. S. *Pseudois nayaur* and *Pseudois schaeferi*. *Mamm. Species* **278**, 1-6 (1987).

99 Tan, S. et al. Molecular evidence for the subspecific differentiation of blue sheep (*Pseudois nayaur*) and polyphyletic origin of dwarf blue sheep (*Pseudois schaeferi*). *Genetica* **140**, 159-167 (2012).

100 Harris, R. B. *Pseudois nayaur*. The IUCN Red List of Threatened Species. 014: e.T61513537A64313015. https://dx.doi.org/10.2305/IUCN.UK.2014-3.RLTS.T61513537A64313015.en (2014).

101 Schaller, G. B. *Wildlife of the Tibetan Steppe* (University of Chicago Press, 1998).

102 Eda, M., Morimoto, M., Mizuta, T. & Inoué, T. ZooMS for birds: Discrimination of Japanese archaeological chickens and indigenous pheasants using collagen peptide fingerprinting. *J. Archaeol. Sci. Rep.* **34**, 102635 (2020).

103 Van Valkenburgh, B. et al. The impact of large terrestrial carnivores on Pleistocene ecosystems. *Proc. Natl. Acad. Sci. USA* **113**, 862-867 (2016).

104 Orlova, L. A., Vasil'ev, S. K., Kuz'min, Y. V. & Kosintsev, P. A. New data on the time and place of extinction of the woolly rhinoceros *Coelodonta antiquitatis* Blumenbach, 1799. *Dokl. Biol. Sci.* **423**, 403-405 (2008).

105 Wang, Y. et al. Late Quaternary dynamics of Arctic biota from ancient environmental genomics. *Nature* **600**, 86-92 (2021).

106 McCarthy, T. M. & Chapron G. *Snow Leopard Survival Strategy* (ISLT and SLN, 2003).

107 Welker, F. et al. The dental proteome of *Homo antecessor*. *Nature* **580**, 235-238 (2020).

108 Andrews P. *Owls, Caves and Fossils: Predation, Preservation, and Accumulation of Small Mammal Bones in Caves, with an Analysis of the Pleistocene Cave Faunas from Westbury-sub-Mendip, Somerset, UK* (University of Chicago Press, 1990).

109 Blumenschine, R. J. Percussion marks, tooth marks, and experimental determinations of the timing of hominid and carnivore access to long bones at FLK Zinjanthropus, Olduvai Gorge, Tanzania. *J. Hum. Evol.* **29**(1), 21-51 (1995).

110 Domínguez-Rodrigo, M. Meat-eating by early hominids at the FLK 22 Zinjanthropus site, Olduvai Gorge, Tanzania: An experimental approach using cut mark data. *J. Hum. Evol.* **33**, 669-690 (1997).

111 Rendu, W. in *Updating Neanderthals: Understanding Behavioural Complexity in the Late Middle Palaeolithic* (eds Romagnoli, F., Rivals, F. & Benazzi, S.) 109-122 (Academic Press, 2022).

112 Blasco, R. et al. in *Updating Neanderthals: Understanding Behavioural Complexity in the Late Middle Palaeolithic* (eds Romagnoli, F., Rivals, F. & Benazzi, S.) 123-143 (Academic Press, 2022).

113 Nilssen, P. J. *An actualistic butchery study in South Africa and its implications for reconstructing hominid strategies of carcass acquisition and butchery in the Upper Pleistocene and Plio-Pleistocene*. Doctoral dissertation. University of Cape Town (2000).

114 Fontana, L., Chauvière, F. X. & Bridault, A. *In Search of Total Animal Exploitation Case Studies from the Upper Palaeolithic and Mesolithic* (BAR International Series 2040, 2009).

115 Johnson, E. & Bement, L. Bison butchery at Cooper, a Folsom site on the Southern Plains. *J. Archaeol. Sci.* **36**, 1430-1446 (2009).

116 Leduc, C. *Acquisition et exploitation des ressources animales au Maglemosien: essai de reconstitution des chaînes opératoires globales d'exploitation d'après l'analyse des vestiges osseux des sites de Mullerup et Lundby Mose (Sjaeland-Danemark)*. Doctoral dissertation. Université Paris 1 Panthéon-Sorbonne (2010).

117 Soulier, M. C. L'exploitation du gibier au Paléolithique supérieur ancien à travers l'exemple des Abeilles (Haute-Garonne, France). *Paléo* **25**, 287-307 (2014).

118 Domínguez-Rodrigo, M. On cut marks and statistical inferences: methodological comments on Lupo and O'Connell (2002). *J. Archaeol. Sci.* **30**, 381-386 (2003).

119 Domínguez-Rodrigo, M. & Pickering, T. R. Early hominid hunting and scavenging: a zooarcheological review. *Evol. Anthropol.* **12**, 275-282 (2003).

120 Pickering, T. R., Domínguez-Rodrigo, M., Egeland, C. P. & Brainc, C. K. Beyond leopards: tooth marks and the contribution of multiple carnivore taxa to the accumulation of the Swartkrans Member 3 fossil assemblage. *J. Hum. Evol.* **46**, 595-604 (2004).

121 Domínguez-Rodrigo, M. et al. Cutmarked bones from Pliocene archaeological sites at Gona, Afar, Ethiopia: implications for the function of the world's oldest stone tools. *J. Hum. Evol.* **48**, 109-121 (2005).

122 Shipman, P. & Rose, J. Early hominid hunting, butchering and carcassprocessing behaviors: approches to the fossil record. *J. Anthropol. Archaeol.* **2**, 57-98 (1983).

123 Stiner, M. C., Barkai, R. & Gopher, A. Cooperative hunting and meat sharing 400-200 kya at Qesem Cave, Israel. *Proc. Natl. Acad. Sci. USA* **106**, 13207-13212 (2009).

124 Zhang, S. Q. et al. Mortality profiles of the large herbivores from the Lingjing Xuchang Man Site, Henan Province and the early emergence of the modern human behaviors in East Asia. *Chin. Sci. Bull.* **54**, 3857-3863 (2009).

125 Zhang, S. Q. et al. Skeletal element distributions of the large herbivores from the Lingjing site, Henan Province, China. *Sci. China Earth Sci.* **55**, 246-253 (2012).

126 Norton, C. J. & Gao X. Hominin-carnivore interactions during the Chinese early paleolithic: taphonomic perspectives from Xujiayao. *J. Hum. Evol.* **55**, 164-178 (2008).

127 Soulier, M. C. & Costamagno S. Let the cutmarks speak! Experimental butchery to reconstruct carcass processing. *J. Archaeol. Sci. Rep.* **11**, 782-802 (2017).

128 Collard, M. et al. Faunal evidence for a difference in clothing use between Neanderthals and early modern humans in Europe. *J. Anthropol. Archaeol.* **44**, 235-246 (2016).

129 d’Errico, F. & Henshilwood, C. S. Additional evidence for bone technology in the Southern African Middle Stone Age. *J. Hum. Evol.* **52**, 142-163 (2007).

130 Sano, K. et al. A 1.4-million-year-old bone handaxe from Konso, Ethiopia, shows advanced tool technology in the early Acheulean. *Proc. Natl. Acad. Sci. USA* **117**, 18393-18400 (2020).

131 Doyon, L., Li, Z., Li, H. & d'Errico, F. Discovery of circa 115,000-year-old bone retouchers at Lingjing, Henan, China. *PLOS ONE* **13**, e0194318 (2018).

132 Rosell, J. et al. Bone as a technological raw material at the Gran Dolina site (Sierra de Atapuerca, Burgos, Spain). *J. Hum. Evol.* **61**, 125-131 (2011).

133 Blasco, R. et al. Using bones to shape stones: MIS 9 bone retouchers at both edges of the Mediterranean Sea. *PLOS ONE* **8**, e76780 (2013).

134 Turner, E. et al. Bone retouchers and technological continuity in the Middle Stone Age of North Africa. *PLoS ONE* **15**, e0230642 (2020).

135 Verna, C. & d’Errico, F. The earliest evidence for the use of human bone as a tool. *J. Hum. Evol.* **60**,145-157 (2011).

136 Baumann, M. et al. On the Quina side: a Neanderthal bone industry at Chez-Pinaud site, France. *PLOS ONE* **18**(6), e0284081 (2023).

137 Villa, P. et al. Elephant bones for the Middle Pleistocene toolmaker. *PLOS ONE* **16**, e0256090 (2021).

138 Kozlikin M. B. et al. Unshaped bone tools from Denisova Cave, Altai. *Archaeol. Ethnol. Anthropol. Eurasia* **48**, 16-28 (2020).
