# Supplementary material for: Middle and Late Pleistocene Denisovan subsistence at Baishiya Karst Cave
Source: Nature. 2024 Jul 3;632(8023):108–13. doi: 10.1038/s41586-024-07612-9 (PMC11291277; doi:10.1038/s41586-024-07612-9)
Supplement: Supplementary file 2 — Reporting Summary [file 41586_2024_7612_MOESM2_ESM.pdf]

nature portfolio

Corresponding author(s): Dr. Frido Welker, Prof. Dongju Zhang, Prof. Fahu Chen

Last updated by author(s): May 21, 2024

Reporting Summary

Nature Portfolio wishes to improve the reproducibility of the work that we publish. This form provides structure for consistency and transparency in reporting. For further information on Nature Portfolio policies, see our Editorial Policies and the Editorial Policy Checklist.

Please do not complete any field with "not applicable" or n/a. Refer to the help text for what text to use if an item is not relevant to your study.
For final submission: please carefully check your responses for accuracy; you will not be able to make changes later.

Statistics

For all statistical analyses, confirm that the following items are present in the figure legend, table legend, main text, or Methods section.

n/a	Confirmed	
☐	☒	The exact sample size (n) for each experimental group/condition, given as a discrete number and unit of measurement
☒	☐	A statement on whether measurements were taken from distinct samples or whether the same sample was measured repeatedly
☐	☒	The statistical test(s) used AND whether they are one- or two-sided<br>*Only common tests should be described solely by name; describe more complex techniques in the Methods section.*
☒	☐	A description of all covariates tested
☒	☐	A description of any assumptions or corrections, such as tests of normality and adjustment for multiple comparisons
☐	☒	A full description of the statistical parameters including central tendency (e.g. means) or other basic estimates (e.g. regression coefficient) AND variation (e.g. standard deviation) or associated estimates of uncertainty (e.g. confidence intervals)
☐	☒	For null hypothesis testing, the test statistic (e.g. F, t, r) with confidence intervals, effect sizes, degrees of freedom and P value noted<br>*Give P values as exact values whenever suitable.*
☐	☒	For Bayesian analysis, information on the choice of priors and Markov chain Monte Carlo settings
☒	☐	For hierarchical and complex designs, identification of the appropriate level for tests and full reporting of outcomes
☒	☐	Estimates of effect sizes (e.g. Cohen's d, Pearson's r), indicating how they were calculated

Our web collection on statistics for biologists contains articles on many of the points above.

Software and code

Policy information about availability of computer code

Data collection	No software was used to collect data.
Data analysis	PEAKS (v. 7.0), mMass (v. 5.5.0), R (v.5.7.1), Geneious Prime (v.2023.2.1), MrBayes (v.3.2.7), RAxML (v.4.0), janitor (v.2.2.0), Biostrings (v.2.68.1), tidyverse (v.2.0.0), ape (v.5.7.1), Vegan (v.2.6-4), MALDIquant (v.1.22.1), MALDIquantForeign (v.0.14).

For manuscripts utilizing custom algorithms or software that are central to the research but not yet described in published literature, software must be made available to editors and reviewers. We strongly encourage code deposition in a community repository (e.g. GitHub). See the Nature Portfolio guidelines for submitting code & software for further information.

Data

Policy information about availability of data

All manuscripts must include a data availability statement. This statement should provide the following information, where applicable:

- Accession codes, unique identifiers, or web links for publicly available datasets
- A description of any restrictions on data availability
- For clinical datasets or third party data, please ensure that the statement adheres to our policy

The mass spectrometry proteomics data have been deposited to the ProteomeXchange Consortium via the PRIDE partner repository with the dataset identifiers PXD041874 and PXD047932. Accession codes for the utilised human reference protein sequences are available in SI Table 4.2 and accessible through Uniprot or Genbank.

Research involving human participants, their data, or biological material

Policy information about studies with human participants or human data. See also policy information about sex, gender (identity/presentation), and sexual orientation and race, ethnicity and racism.

Reporting on sex and gender	NA
Reporting on race, ethnicity, or other socially relevant groupings	NA
Population characteristics	NA
Recruitment	NA
Ethics oversight	NA

Note that full information on the approval of the study protocol must also be provided in the manuscript.

Field-specific reporting

Please select the one below that is the best fit for your research. If you are not sure, read the appropriate sections before making your selection.

☐ Life sciences ☐ Behavioural & social sciences ☒ Ecological, evolutionary & environmental sciences

For a reference copy of the document with all sections, see nature.com/documents/nr-reporting-summary-flat.pdf

Ecological, evolutionary & environmental sciences study design

All studies must disclose on these points even when the disclosure is negative.

Study description	Proteomic analysis and zooarchaeological analysis for bone specimens.
Research sample	Animal bones and teeth.
Sampling strategy	We selected almost all available faunal specimens longer than 20 mm, as well as some smaller skeletal fragments (under 20 mm) with morphological characteristics suitable for traditional zooarchaeological analysis. Further details can be found in the Methods section.
Data collection	All specimens were derived from archaeological excavations conducted within Baishiya Karst Cave by Lanzhou University and Gansu Provincial Institute of Cultural Relics and Archaeological Research.
Timing and spatial scale	Middle and Late Pleistocene, northeast Tibetan Plateau. Further details can be found in the SI document.
Data exclusions	Data was not excluded during any part of the study. Where possible, subsets of data were included for specific comparative analyses, as explained in the Methods section, SI document, or figure/table legends (wherever applicable).
Reproducibility	Our study includes cross-validation of taxonomic identifications by performing different (proteomic) methods for some faunal specimens and proteomic extracts, as indicated in the manuscript.
Randomization	Randomization was not relevant to this study.
Blinding	Blinding was not relevant to this study.

Did the study involve field work? ☒ Yes ☐ No

Field work, collection and transport

Field conditions	High-altitude environments.
Location	Xiahe county, China. 35.45° N, 102.57° E
Access & import/export	The excavations are permitted by the National Cultural Heritage Administration, China (2018-246, 2019-551).

Disturbance	None.

Reporting for specific materials, systems and methods

We require information from authors about some types of materials, experimental systems and methods used in many studies. Here, indicate whether each material, system or method listed is relevant to your study. If you are not sure if a list item applies to your research, read the appropriate section before selecting a response.

Materials & experimental systems

n/a	Involved in the study	
☒	☐	Antibodies
☒	☐	Eukaryotic cell lines
☐	☒	Palaeontology and archaeology
☒	☐	Animals and other organisms
☒	☐	Clinical data
☒	☐	Dual use research of concern
☒	☐	Plants

Methods

n/a	Involved in the study	
☒	☐	ChIP-seq
☒	☐	Flow cytometry
☒	☐	MRI-based neuroimaging

Palaeontology and Archaeology

Specimen provenance	Faunal specimens from Baishiya Karst Cave were collected from archaeological excavations in 2018 and 2019, the details can be found in the Methods and SI sections.
Specimen deposition	Faunal specimens, the details can be found in the Methods and SI sections.
Dating methods	The study conducts no novel dating attempts on Baishiya Karst Cave. Reference is made to previous publications providing chronological data on this, and other, archaeological and palaeoanthropological sites.

☒ Tick this box to confirm that the raw and calibrated dates are available in the paper or in Supplementary Information.

Ethics oversight	There are no ethical issues involved in this study.

Note that full information on the approval of the study protocol must also be provided in the manuscript.

Plants

Seed stocks	NA
Novel plant genotypes	NA
Authentication	NA

This checklist template is licensed under a Creative Commons Attribution 4.0 International License, which permits use, sharing, adaptation, distribution and reproduction in any medium or format, as long as you give appropriate credit to the original author(s) and the source, provide a link to the Creative Commons license, and indicate if changes were made. The images or other third party material in this article are included in the article's Creative Commons license, unless indicated otherwise in a credit line to the material. If material is not included in the article's Creative Commons license and your intended use is not permitted by statutory regulation or exceeds the permitted use, you will need to obtain permission directly from the copyright holder. To view a copy of this license, visit http://creativecommons.org/licenses/by/4.0/
